# Additive-Driven Interfacial Engineering of Aluminum Metal Anode for Ultralong Cycling Life

**DOI:** 10.1007/s40820-022-01000-6

**Published:** 2022-12-29

**Authors:** Sonal Kumar, Prasad Rama, Gaoliang Yang, Wei Ying Lieu, Deviprasath Chinnadurai, Zhi Wei Seh

**Affiliations:** 1 Institute of Materials Research and Engineering, Agency for Science, Technology and Research (A*STAR), 2 Fusionopolis Way, Innovis, Singapore, 138634 Singapore; 2 Department of Chemistry and Molecular Biology, University of Gothenburg, 41125 Gothenburg, Sweden; 3 Pillar of Engineering Product Development, Singapore University of Technology and Design, 8 Somapah Road, Singapore, 487372 Singapore

**Keywords:** Aluminum-ion batteries, Solid electrolyte interphase, Electrolyte additives, Non-aqueous electrolytes

## Abstract

Rechargeable Al batteries (RAB) are promising candidates for safe and environmentally sustainable battery systems with low-cost investments. However, the currently used aluminum chloride-based electrolytes present a significant challenge to commercialization due to their corrosive nature. Here, we report for the first time, a novel electrolyte combination for RAB based on aluminum trifluoromethanesulfonate (Al(OTf)_3_) with tetrabutylammonium chloride (TBAC) additive in diglyme. The presence of a mere 0.1 M of TBAC in the Al(OTf)_3_ electrolyte generates the charge carrying electrochemical species, which forms the basis of reaction at the electrodes. TBAC reduces the charge transfer resistance and the surface activation energy at the anode surface and also augments the dissociation of Al(OTf)_3_ to generate the solid electrolyte interphase components. Our electrolyte's superiority directly translates into reduced anodic overpotential for cells that ran for 1300 cycles in Al plating/stripping tests, the longest cycling life reported to date. This unique combination of salt and additive is non-corrosive, exhibits a high flash point and is cheaper than traditionally reported RAB electrolyte combinations, which makes it commercially promising. Through this report, we address a major roadblock in the commercialization of RAB and inspire equivalent electrolyte fabrication approaches for other metal anode batteries.

**Supplementary Information:**

The online version contains supplementary material available at 10.1007/s40820-022-01000-6.

## Highlights

- A unique electrolyte combination for rechargeable aluminum battery was fabricated using aluminum trifluoromethanesulfonate as the main salt and tetrabutylammonium chloride as an additive.
- The presence of additive activates the Al-anode surface toward plating/stripping and aids in the formation of a protective solid electrolyte interphase layer on the anode, resulting in a substantially suppressed anodic overpotential for 1300 cycles.
- The cheap, high flash point and non-corrosive nature of the electrolyte makes it commercially promising.

**Supplementary Information:**

The online version contains supplementary material available at 10.1007/s40820-022-01000-6.

## Graphical abstract

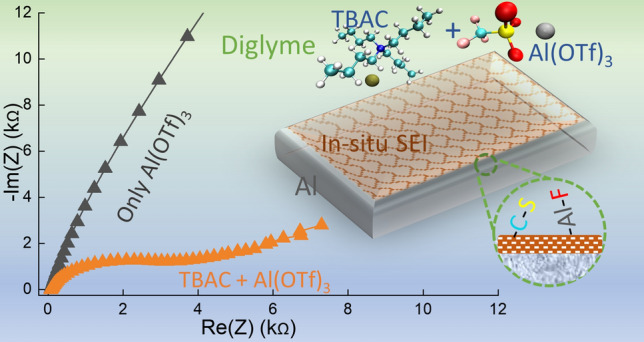

## Introduction

The increasing demand for electric vehicles, aided by the unequal geographical distribution of lithium reserves, is likely to keep the supply of lithium tight and increase the production cost of Li-ion batteries [1]. A tight Li supply–demand balance is expected in the near future unless new supply lines come online, which may have undesirable socio-environmental impacts once full-fledged lithium mining is underway [2]. Hence, the uncertainty in the lithium supply is pushing the battery research and development toward non-lithium chemistries, which utilize earth-abundant metals, including Na, K, Mg, Ca, Zn and Al [3–10]. Among all, Al-metal chemistry is the most promising because of: (1) highest abundance (among metals) in the earth’s crust, (2) highest volumetric capacity (~ 8000 mAh cm^−3^) and (3) economically sustainable raw material supply because of an already matured Al industry [11]. On top of that, most of the reported Al-salt-based electrolytes have higher flash point (stable in ambient environment [12]) than currently commercialized Li-ion battery electrolytes (flash point ~ 25 °C), [13] making the battery inherently safe at room temperature and opening avenues for: (1) battery assembly in less stringent environmental conditions, (2) battery operation at higher temperatures and (3) reduction in safety measures like battery management systems. Consequently, there has been renewed interest in the exploration of rechargeable Al battery (RAB) chemistry [14].

However, RAB research being in a relatively nascent stage, has its specific limitations. In the case of electrolyte systems, the most commonly reported non-aqueous electrolyte for RAB, anhydrous AlCl_3_ + ionic liquid (1-ethyl-3-methylimidazolium chloride (EMICl) or 1-butyl-3-methylimidazolium chloride), is undesirably expensive and corrosive in nature, limiting its usage at large scale [15]. Though some cheaper ionic liquid analog alternatives like AlCl_3_ + urea and AlCl_3_ + triethylamine hydrochloride have been reported [16], the use of AlCl_3_ still makes the electrolytes corrosive. Researchers have also attempted to plate/strip Al using haloaluminate-free electrolytes but with limited success. For instance, unsuccessful plating/stripping was reported for aluminum trifluoromethanesulfonate (Al(OTf)_3_) in diglyme and *N*-methyl acetamide/urea, even though Al-ion was found to be active in the electrolytes [17–19]. Later, Al electrodeposition was attempted for aluminum 1-butylimidazole bis(trifluoromethanesulfonyl)imide which resulted in quasi-reversible plating/stripping (reversible only for few cycles or with excessive side reactions) [20]. Similar quasi-reversible behavior with large plating/stripping overpotential (> 1.5 V) was observed for Al(TFSI)_3_ in acetonitrile and Al(PF_6_)_3_ in dimethyl sulfoxide [21, 22]. Irreversible Al electrodeposition was also reported for Al(OTf)_3_ in tetrahydrofuran [23, 24]. Other electrolyte alternatives, including molten salts, gel polymer electrolytes and hybrid electrolytes, have been demonstrated as well [15]. However, they come with additional requirements of high operation temperature, incompatibility with existing fabrication facility and requirement of lithium-containing cathodes, respectively [15].

There are mainly two approaches to achieving successful Al plating/stripping: an extrinsic approach which includes anode modifications outside the cell and then using the modified anode for cycling, such as anode amorphization [25], anode alloying [26] and using a composite anode [27]. Alternatively, there is an intrinsic approach, which includes electrolyte modification in the cell, such as the fabrication of concentrated electrolytes, [28] adding scavengers [29] and additives into the electrolyte [30]. An intrinsic approach is advantageous, as the problem of corrosion, as well as plating/stripping, can be tackled at once by modifying the electrolyte. Very interestingly, all the electrolytes mentioned above which show reversible plating/stripping use corrosive AlCl_3_ as the main salt, implying the importance of Cl^−^ ion in the electrolyte, either to form charge carrying complexes or to break the passivation layer on the Al-metal anode [31, 32]. Based on the above discussion, a promising developmental step for RAB is the exploration of new electrolyte configurations which are cheap, non-corrosive and preferably contain small amounts of Cl^−^ ion.

In this work, we report for the first time, a novel electrolyte configuration for RAB that is based on Al(OTf)_3_ with tetrabutylammonium chloride (TBAC) additive in diglyme. This electrolyte does not comprise corrosive AlCl_3_ as the main electrolyte salt; instead, we only use a small amount (0.1 M) of organic additive, TBAC, with Al(OTf)_3_ as the main salt (0.5 M) dissolved in diglyme. The advantage of using TBAC is first seen in terms of high reversibility and reduced plating/stripping overpotential on the Al anode surface. Further, using a combination of electrochemical and material characterization studies, we decipher that the addition of TBAC in the electrolyte: (1) generates the required speciation, Al_2_Cl_7_^−^ and AlCl_4_^−^, for charge transfer to proceed on the electrodes, (2) helps in rendering the passivated Al surface active toward plating/stripping as seen in terms of reduced charge transfer resistance and reduced surface activation energy at the Al metal anode and (3) causes enhanced dissociation of Al(OTf)_3_ to generate free OTf^−^ ions which further decomposes and forms a robust solid electrolyte interphase (SEI) layer on the anode, critical in protecting the Al anode from oxidation during cycling. Molecular dynamics (MD) is a potential tool to evaluate the inter atomic/ionic/molecular interactions among the constituents of the electrolyte to get an insight into the complexation among ions/molecules [33–35]. Using MD simulation and extensive surface characterization, respectively, we also elucidate the changes occurring in the solvation sheath of Al-ions upon the addition of TBAC and the chemical composition of the SEI layer as a function of depth. Finally, we validate the use case of our additive modulated electrolyte by demonstrating reversible intercalation in an Al metal-graphite full-cell.

## Experimental Methods

### Battery Fabrication and Testing

High-purity Al foil (0.25 mm (0.01 in) thick, annealed, 99.99% (metals basis), CAS: 7429-90-5) was first punched in the form of circular disks, polished with sandpaper of grit size 2000 inside a glovebox (O_2_ < 1.2 ppm and H_2_O < 0.1 ppm) and then either used in a symmetric cell or as an anode in an asymmetric cell. For electrolyte fabrication, diglyme solvent (Sigma Aldrich, CAS: 111-99-6, anhydrous, 99.5%) was first dried overnight using molecular sieves and further added to various mixtures of Al(OTf)_3_ salt (Sigma Aldrich, CAS: 74974-61-1, 99.9% trace metals basis) and TBAC additive (Sigma Aldrich, CAS: 1112-67-0, > 97.0%) as per the requirement. The electrolyte mixture was left to stir at least for 4 h without any application of heat. Notably, the low concentration electrolytes (0.1 M and 0.5 M Al(OTf)_3_) dissolved giving a clear solution; however, some high concentration electrolyte showed some precipitated salt when higher amounts of TBAC was added in them (1 M and 2 M Al(OTf)_3_ with 0.5 M TBAC; Fig. S1a). An oversaturated diglyme-Al(OTf)_3_ solution which did not dissolve completely gave clearer solution when TBAC was added in it (Fig. S1a right part).

For the cathode preparation, commercially obtained expanded graphite (EG 500, expanding rate of 500 at 1237 K treating, Hangzhou Gaoxi Technology Co. Ltd., China) was pressed into the form of tablets using a press, dried at 60 °C overnight and then used as cathode. It can be noted here that the adhesive nature of EG500 enables us to use them as free-standing film (~ 22 mg; 0.5 mm thick), hence getting rid of the current collector as well as binder. Finally, battery fabrication was done inside an argon-filled glovebox (O_2_ < 1.2 ppm and H_2_O < 0.1 ppm) in a 2032-coin cell with 80 μL of electrolyte soaked on the glass fiber separator (Whatman™, GF/A; dried at 150 °C for at least 4 h).

Further, fabricated cells were rested for at least 5 h before electrochemical characterizations. Gamry 3000 or Gamry 1010 potentiostat was used for cyclic voltammetry (CV), linear sweep voltammetry (LSV) and electrochemical impedance spectroscopy (EIS) studies. CV for plating/stripping study was done in a 3-electrode setup with Pt as working electrode and Al foil as counter- and pseudoreference electrode. CV for the full-cell was done in a coin-cell setup. For each EIS measurement, 10 frequency points were distributed logarithmically per decade in the range 100 kHz to 0.01 Hz with an amplitude voltage of 5 mV. Temperature-dependent EIS was done from 25 to 70 °C, with cells kept in a temperature-controlled chamber. BTS Neware Battery Testing System was used for plating/stripping and galvanostatic charge–discharge (GCD) studies. Plating stripping study was done at constant or different current densities (*x* mA cm^−2^, *x* mAh cm^−2^) with each plating or stripping step occurring for 1 h. EG500 was charged/discharged by cycling (CV) it from OCV to 2.5 V and back to 0.5 V (or 2.7 to 0.3 V) in a coin cell using Al as anode. For characterization purposes, the charged and discharged EG500 was held for > 10 h at 2.5 and 0.5 V, respectively.

### Materials Characterization

A Renishaw Invia Confocal Raman spectrometer equipped with 785-nm argon laser and 532-nm argon laser was used to measure the Raman shifts for the electrolytic liquids and EG500 cathodes, respectively. FESEM was conducted using JEOL 7600F operated at 5 keV to visualize surface of Al anode and EG500 cathode. EDX analysis was done using a higher potential bias of 15 keV. Thermo Scientific theta probe angle-resolved spectrometer (XPS) equipped with 1486.69 eV, Al Kα radiation was used to investigate the surface of the Al foil and cathode. A customized XPS holder was used to transfer the anode samples from the glovebox directly to XPS chamber, without exposing the samples to the ambient environment. Cathode samples were opened in the air. XPS depth profiling was conducted using Ar^+^ sputtering at 3.0 keV, 2 μA (etching rate of 2.5 nm min^−1^ on Si wafer) by etching an area of 4 × 4 mm^2^ area. The signal collection was from a circular area of diameter 400 μm, to ensure that the signals were collected only from the etched area. For narrow scan, pass energy of 40 eV with a step size of 0.1 eV was used. For the survey spectrum, pass energy of 200 eV with a step size of 1 eV was used. Peak deconvolution was done in CASA XPS software using Shirley background and a combination of Lorentzian & Gaussian distribution (30:70) model peak. All the data were calibrated to 284.8 eV, owing to the presence of adventitious carbon. Electrolyte conductivity measurement was done using Fisherbrand Accumet AB200. Flash point measurements were done using a Setaflash Series 8 closed cup flashpoint tester.

### Simulation Methodology

Molecular dynamics (MD) simulation is a potential tool to evaluate the interactions among the atoms/molecules/ions at an atomistic level. Three independent simulations including 0.5 M Al(OTf)_3_ + 0 M TBAC, 0.5 M Al(OTf)_3_ + 0.05 M TBAC and 0.5 M Al(OTf)_3_ + 0.1 M were considered in this study to evaluate the interactions among Al(OTf)_3_, TBAC and diglyme. The simulation box considered in this work is 4 × 4 × 4 nm^3^ in size with periodicity in all three dimensions replicating an infinite system. The simulation box is first filled up with the solvent molecules diglyme, and then, an appropriate number of salt molecules, Al(OTF)_3_ or additive molecules, TBAC are introduced randomly into the simulation box by replacing the solvent diglyme molecules. The snapshot representing the constituents of a simulation box is shown in Fig. S6a.

All the MD simulations were performed using GROMACS 2021.5 [36] software package and CHARMM (Chemistry at Harvard Macromolecular Mechanics) force fields. The initial structural coordinates and the topologies of all the required molecules for the simulation were generated utilizing CHARMM-GUI [37, 38]. The initial structures were energy minimized and equilibrated well in two stages, with an NVT and an NPT ensemble maintaining a temperature and pressure of 300 K and 1 atm, respectively, for about 100 ps each. A Berendsen [39] thermostat and a barostat with a relaxation time of *τ* = 0.1 ps and *τ* = 2.0 ps were used to maintain the temperature and pressure of the simulation box. As the system attains equilibration in temperature and pressure, the final production runs were performed using an NVT ensemble for 50 ns using 2 fs as a time step for integrating the equations of motion. All the radial distribution profiles reported in this work were calculated using a bin width of 0.05 nm radially outward in direction starting from the center of mass of Al^3+^ ion toward the selected molecule (considered as a group).

## Results and Discussion

### Anodic Plating/Stripping

To investigate the additive to salt ratio, a series of Al(OTf)_3_ as main Al salt and TBAC as the electrolyte additive, with varied molar configurations, were prepared in diglyme as the solvent. The digital images of the prepared electrolytes are shown in Fig. S1a. Notably, an oversaturated Al(OTf)_3_-diglyme solution, which did not dissolve completely, gave a clearer solution when TBAC was added to it (Fig. S1a, right part), providing initial indications that TBAC might assist in improving the solubility of Al(OTf)_3_.

A series of galvanostatic plating/stripping studies were done in the above-mentioned electrolyte configurations to achieve reduced anodic overpotential. Firstly, the Al(OTf)_3_ concentration was optimized for a fixed concentration of TBAC (Fig. S1b). The plating/stripping indicates least overpotential for 0.5 M Al(OTf)_3_, the reason for which can be its high ionic conductivity (Fig. S3b). Further, the concentration of TBAC was varied and optimized for fixed concentrations of Al(OTf)_3_ in the diglyme (Fig. S1c–e). Interestingly, the least anodic overpotential was observed for 0.1 M TBAC in all three concentrations (0.25, 0.5 and 1 M) of Al(OTf)_3_. Among all, 0.5 M Al(OTf)_3_ + 0.1 M TBAC showed the least overpotential at 0.1 mA cm^−2^, which was also sustained at a higher current density of 1 mA cm^−2^ (Fig. S1d).

**Fig. 1:**
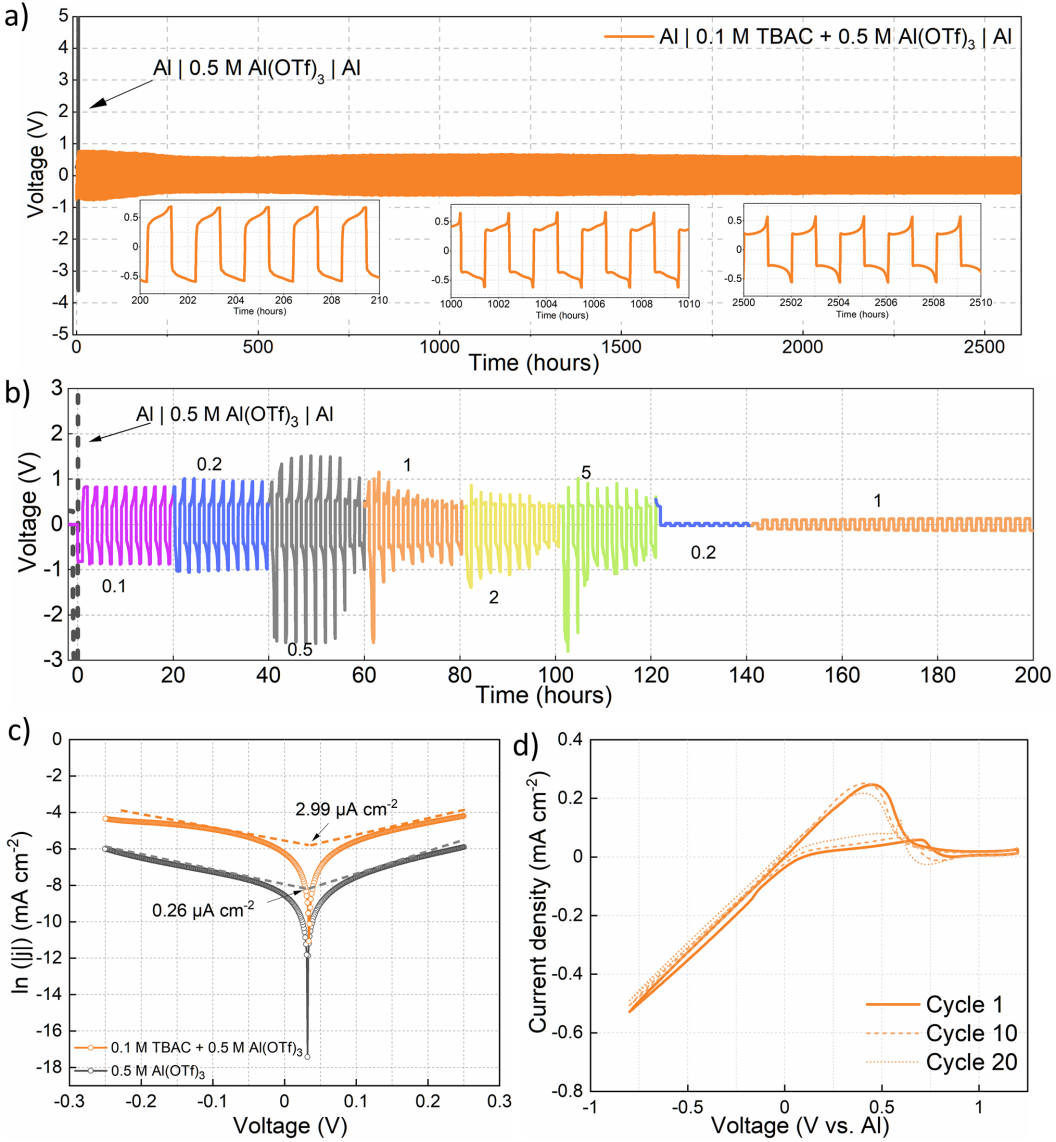
Comparative plating/stripping study in 0.5 M Al(OTf)_3_ vs. 0.5 M Al(OTf)_3_ + 0.1 M TBAC at: **a** 0.1 mA cm^−2^, 0.1 mAh cm^−2^ and **b** multiple current density, *x*, as indicated in Fig. (*x* mA cm^−2^, *x* mAh cm^−2^). **c** Tafel curves and corresponding exchange current densities from the linear sweep voltammetry studies (−0.25 to 0.25 V at 1 mV s^−1^) in two electrolytes, 0.5 M Al(OTf)_3_ and 0.5 M Al(OTf)_3_ + 0.1 M TBAC. **d** CV scan at 20 mV s^−1^ in 0.5 M Al(OTf)_3_ + 0.1 M TBAC electrolyte with Pt as working electrode, and Al as counter- and pseudoreference electrode

A comparative plating/stripping study done with and without TBAC revealed a ten times reduction in the overpotential when TBAC is added in a small concentration (0.1 M) in the electrolyte (Fig. 1a). While the TBAC absent electrolyte (0.5 M Al(OTf)_3_; represented by the gray line) showed a very high overpotential of 5 V, the optimized configuration of electrolyte (0.5 M Al(OTf)_3_ + 0.1 M TBAC) showed ~ 0.5 V overpotential. Clearly, the addition of TBAC in the electrolyte is responsible for activating the Al surface, further resulting in lower platting/stripping overpotential. Most interestingly, the anodic overpotential decreased to ~ 0.4 V as the cycling proceeded (three insets in Fig. 1a) over an ultralong cycling life of 1300 cycles (2600 h).

A similar decrease in the overpotential was observed more clearly when plating/stripping at multiple current density from 0.1 to 5 mA cm^−2^ (Figs. 1b and S1f). Figure 1b shows a tenfold reduction in the overpotential to < 0.1 V when the cycling current density was reduced to 0.2 mA cm^−2^ after 60 cycles (120 h) of plating/stripping at various current density. The cell was still able to cycle reversibly when the current density was finally increased to 1 mA cm^−2^ (hence also indicating that there is no short-circuiting). Such decreasing overpotential with repeated cycling has been observed for other systems as well and is likely due to the slow activation of the anode surface, which can occur for various reasons, including breaking of the native oxide layer and removal of surface impurity/electrochemically inactive species [29, 30]. We want to note here that the overpotential decrease during the plating/stripping at constant current density of 0.1 mA cm^−2^ (Fig. 1a) is not as prominent as when switching the current density from 5 to 0.2 mA cm^−2^ in the multi-current density plating/stripping (Fig. 1b). The primary reason for this difference can be the different extent of surface area activated at different current densities. For example, when cycling at a higher current density, there are likely more spots where the oxide layer is broken (as a higher current density of plating leads to higher overpotential, which leads to the formation of a more significant number of nucleation sites) [40, 41]. Hence, there is an increased amount of area activated at high current density. But when switching the current back to a lower current density, the effective area remains high, while the current amplitude is the same. This would consequently lead to reduced current per unit area, and hence, there is reduced overpotential after the current density switch. Our group observed this exact same phenomenon for Mg symmetric cells in the past [30].

We also note the presence of some overpotential spikes when cycling the symmetric cell at multiple current densities. It is known that Al plating/stripping is unlike other plating/stripping mechanism, which merely involves deposition and removal of the metal ion [12]. For Al, the chlorinated Al (and not Al^3+^) gets deposited and removed from the anode. Hence, at times when plating would not have occurred 100 percent, there can be some overpotential spikes during the stripping process (because the Al metal anode cannot provide chlorinated Al). These random spikes occur more often when cycling the symmetric cells at multiple current densities because of the surface activation phenomenon discussed above. Therefore, the random spikes, as shown in Fig. 1b, should be treated as an experimental artifact. It can be well noted here that when cycling the symmetric cell at a constant current density of 0.5 mA cm^−2^ (Fig. S1g), unwarranted spikes in the overpotential (as was observed at 0.5 mA cm^−2^ in Fig. 1b) are not observed, rather the overpotential remains stable throughout cycling.

The plating/stripping study is also supported by the Tafel curves derived from the linear sweep voltammetry measurements (Fig. 1c). A higher exchange current density (2.99 vs 0.26 µA cm^−2^) is obtained for the TBAC modulated Al(OTf)_3_ electrolyte as against pure Al(OTf)_3_, indicating that the Al anode surface is reacting more readily in the presence of TBAC [42]. Here, we would like to highlight that the exchange current density mostly corresponds to the Al species deposition during plating, as seen from the deposited Al on the surface of Ni foil in 0.5 M Al(OTf)_3_ + 0.1 M TBAC (Fig. S2a).

Further, cyclic voltammetry (CV) study of 0.5 M Al(OTf)_3_ + 0.1 M TBAC on Pt (working electrode) shows reversible plating/stripping (Fig. 1d). The cathodic scan displays a reductive current starting from 0 V associated with Al plating and similarly, the anodic scan displays an oxidative current associated with Al stripping. We also note that our plating/stripping is highly recurring as shown by nearly overlapping 1st, 10th and 20th cycling curves. Reversible and recurring plating/stripping with low overpotential (< 0.5 V) makes our electrolyte combination superior to previously reported non-AlCl_3_ organic electrolytes for RAB. These previous reports showed unsuccessful [17–19], irreversible, [23, 24] and/or quasi-reversible [20–22] CV curves, which limits the practical application of such electrolytes. Further, we also point out one specific work by Reed et al. wherein they did CV (with Pt working electrode) of various concentrations of Al(OTf)_3_ in diglyme [18]. Notably, they did not observe any plating/stripping and attributed the unsuccessful plating/stripping to the passivation of Al anode during cycling [18]. Comparing our CV curve with Reed’s work [18], the crucial role of TBAC additive in regulating the Al surface chemistry becomes clear. We speculate that the presence of TBAC directly or indirectly helps in breaking the passivation layer on Al and later also maintains the surface chemistry active throughout the cycling. Notably, our approach to achieving successful plating/stripping is of an intrinsic modification in the electrolyte and different from extrinsic modification approaches like anode amorphization [25], anode alloying [26] and usage of composite anodes [27].

### Electrolyte Superiority

Further superiority of our electrolyte is collectively reflected in the flash point study, the corrosion study and the price comparison with the reported non-aqueous RAB electrolytes. Closed cup flash point testing of various concentrations of TBAC in 0.5 M Al(OTf)_3_-diglyme electrolyte reveals a flashpoint in the range of 57 to 60 °C (Table S1). A flashpoint of 60 °C is significantly higher than that of most commonly used Li-ion battery electrolytes at a large scale [13, 43]. It opens up the opportunity to make safer battery systems under lesser stringent battery fabrication conditions. For corrosion study, extensive visual examination of the coin cell parts was done for cells rested for 1 month, and cycled for 62 and 600 cycles. No signs of corrosion were detected in any of the cases (Fig. S2b). SEM analysis of stainless steel (SS—spacer used in coin cell) after dipping it in ~ 15 mL of 0.5 M Al(OTf)_3_ + 0.1 M TBAC for ~ 60 h revealed negligible signs of corrosion (Fig. S2c, d), as compared to the large corrosion pits typically observed in AlCl_3_-based electrolytes [44]. Chronoamperometry on Ti, SS, Ni and Mo (Fig. S2e–h, respectively) also supports substantially reduced corrosion in 0.5 M Al(OTf)_3_ + 0.1 M TBAC electrolyte vs. commercial (Sigma Aldrich—CAS 742872) AlCl_3_ + EMICl (3:2) electrolyte. A reduced leakage current was observed on Ti, SS and Ni in our electrolyte when the metals were subjected to different potentials values from 1 to 2.6 V. Notably, for the case of SS and Ni in AlCl_3_ + EMICl, there was a sharp increase in the leakage current to ~ 10 mA cm^−2^ when the voltage was increased to 1.5 V. For the case of Mo, the leakage current in both the electrolytes remained low. Chronoamperometry study reveals that there are suppressed side reactions happening for our TBAC-based electrolyte as compared to the traditional AlCl_3_ + EMICl electrolyte, which is also indicative of a substantially reduced corrosion, if any. Further, price comparison of electrolytes reveals our electrolyte to be ~ 5 times cheaper than conventional EMICl + AlCl_3_ eutectic electrolyte and ~ 4 times cheaper than eutectic analogue, urea + AlCl_3_ electrolyte (Table S2). Thus, beyond the fact that a lower overpotential can be achieved in our electrolyte, its high flashpoint, corrosion-free nature and cost-effectiveness make our electrolyte extremely promising for commercial production.

### Anode–Electrolyte Interface

**Fig. 2:**
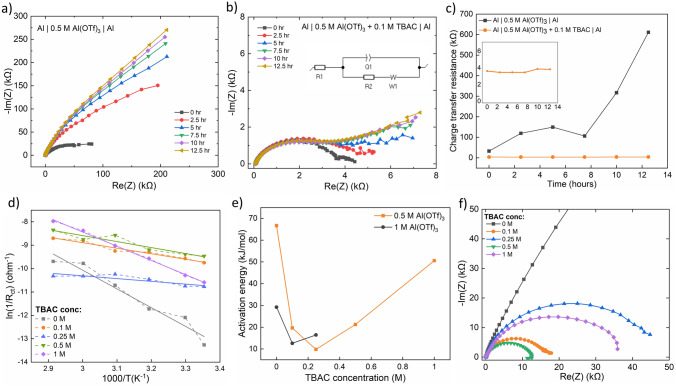
Nyquist plot collected at 2.5 h of intervals after cell fabrication for: **a** Al | 0.5 M Al(OTf)_3_ | Al symmetric cells, **b** Al | 0.5 M Al(OTf)_3_ + 0.1 M TBAC | Al symmetric cells (inset: circuit used for fitting a and b). **c** Charge transfer resistance as a function of time, generated after fitting impedance data in Fig. 2a, b (inset: zoomed in data for TBAC cell). **d** Logarithmic plot of inverse of charge transfer resistance (obtained after fitting respective data in Fig. S4) versus the inverse of the temperature. Each plot, which is also linearly fitted with the solid lines, is for Al | 0.5 M Al(OTf)_3_ + *x* M TBAC | Al symmetric cell with varying concentration (*x*) of TBAC. **e** Surface activation energy as a function of TBAC concentration (calculated from Figs. 2d and S5d for 0.5 and 1 M Al(OTf)_3_ electrolyte, respectively). **f** Nyquist plot collected for Al | 0.5 M Al(OTf)_3_ + *x* M TBAC | Al symmetric cells with varying concentration (*x*) of TBAC

Further, a detailed EIS study carried on Al symmetric cells at rest, during cycling and at varying temperature, reveals the advantages of using TBAC with the Al(OTf)_3_ electrolyte and provides critical insights into why 0.5 M Al(OTf)_3_ + 0.1 M TBAC configuration performs the best. EIS study done at intervals of 2.5 h from the time the cell was fabricated shows significantly different impedance when using Al(OTf)_3_ vs. 0.1 M TBAC + Al(OTf)_3_ electrolyte (Fig. 2a vs. b). The overall impedance for the Al(OTf)_3_ cell starts from about 100 kΩ and increases to a larger value with resting time. However, for the TBAC modulated electrolyte, the initial impedance is about 1/20th (5 kΩ) of the former and does not increase significantly during the 12.5 h of rest. Charge transfer resistance (*R*_ct_), obtained after fitting the circuit (inset of Fig. 2b) to the EIS data of the electrolytes, plotted with the resting time, reveals a constantly low *R*_ct_ value for the TBAC modulated electrolyte, whereas the *R*_ct_ keeps on increasing for the non-modulated electrolyte (Fig. 2c). From these observations, we infer that the presence of TBAC is not only activating the Al anode surface in the initial stage of the cell fabrication but also aiding in maintaining its activity through later stages. This low impedance is likely due to the Cl^−^ ion from TBAC which assisted in breaking the oxide layer on the Al surface [6, 31]. Further, the distinct dynamically changing nature of the EIS spectra, when comparing both the cases of electrolyte, indicates an imperative role of TBAC in regulating the surface reactions. Notably, for the case of Al(OTf)_3_, the spectra reveal only a kinetically controlled regime throughout the 12.5 h of rest, indicating an anode-only controlled reaction at the surface. However, for TBAC + Al(OTf)_3_, a kinetically controlled charge transfer regime, seen at 0 h of rest, develops into a kinetic + diffusion-controlled regime during the 12.5 h of rest, signifying that both the electrolyte and the anode surface are controlling the charge transfer at the surface. Further, during the cycling, the overall impedance for the TBAC + Al(OTf)_3_ remained in the same order as at the 12.5th hour (Fig. S3a), indicating that the anode surface is well stabilized and protected even after repeated cycling. The non-dendritic nature of the Al deposits is also indicated from the SEM study of the cycled Al anode (Fig. S3c–f). After 20 cycles, the surface morphology of the Al anode changes from flat to rugged, possibly due to in-situ SEI formation along with Al complex deposition.

Temperature-based EIS study was done systematically for a series of electrolytes, which revealed important information in terms of surface activation energy (*E*_*a*_) in various electrolytes. Reciprocal of *R*_ct_, i.e., conductance, is expected to follow an Arrhenius equation as follows:

1 $$\frac{1}{{R}_{\mathrm{Ct}}}=A{\mathrm{e}}^{-\frac{{E}_{a}}{{k}_{B}T}}$$

where *E*_a_ is the surface activation energy of ions moving in the surface layer, k_B_ is the Boltzmann constant, *T* is the temperature and A is the proportionality constant [45]. *E*_a_ was obtained from the EIS study done from 25 to 70 °C. Firstly, temperature-based EIS study was done for 0.5 M Al(OTf)_3_ with varying amounts of TBAC (Fig. S4). After fitting the spectra in Fig. S4 and generating a temperature-dependent *R*_ct_ chart (Fig. 2d), *E*_a_ was obtained as a function of TBAC concentration. Very interestingly, the activation energy first decreases and then increases with the TBAC concentration (*E*_a_: 0.25 M < 0.1 M < 0.5 M < 1 M < 0 M) (Fig. 2e), revealing that 0.25 M might be the ideal concentration of TBAC to be used from a standpoint of low surface activation energy. However, the EIS spectra at 25 °C for varying amount of TBAC (Fig. 2f) reveal the following *R*_ct_ order: 0.5 M < 0.1 M < 1 M < 0.25 M < 0 M, indicating that 0.5 M might be the best choice from a standpoint of low *R*_ct_. However, considering both the factors *E*_a_ and *R*_ct_, and the fact that we want to use a minimum amount of Cl^−^ in the electrolyte to avoid corrosion, 0.1 M TBAC is the ideal choice. The choice of 0.1 M TBAC also corroborates with our plating/stripping study, wherein the lowest overpotential was observed when using 0.1 M additive (irrespective of Al(OTf)_3_ concentration).

To enhance our understanding of varying *E*_a_ with TBAC concentration, another set of temperature-based EIS study was done, this time for different amounts of TBAC in 1 M Al(OTf)_3_ (Fig. S5). Similar to the case of 0.5 M Al(OTf)_3_, the *E*_a_ first decreases and then increases with the TBAC concentration (black line in Fig. 2e). This pattern bolsters our belief that TBAC concentration optimization is crucial to our study and hence our extended efforts for the same. Further, conductivity study (Fig. S3b) reveals dependence on both Al(OTf)_3_ and TBAC concentration. Conductivity increases and then decreases with increasing concentration of Al(OTf)_3_, with maximum conductivity observed for 0.5 M Al(OTf)_3_, making it the preferred choice. At the same time, conductivity increases linearly with the TBAC concentration. However, we speculate that the activation energy dependence on TBAC concentration supersedes the choice of using high concentration TBAC for better ionic conductivity advantage.

From our detailed EIS analysis, we conclude that the addition of TBAC in the electrolyte not only brings down the impedance by a factor of 20 but also aids in maintaining it at a low value during rest as well as during cycling, clearly showing the advantages of using TBAC. Most importantly, we also infer that an optimized amount of TBAC, here 0.1 M, in a highly conducting electrolyte, here 0.5 M Al(OTf)_3_, is critical in obtaining the right combination of *E*_*a*_ and *R*_ct_, which eventually results in lower values of plating/stripping overpotential.

### Electrolyte Speciation

**Fig. 3:**
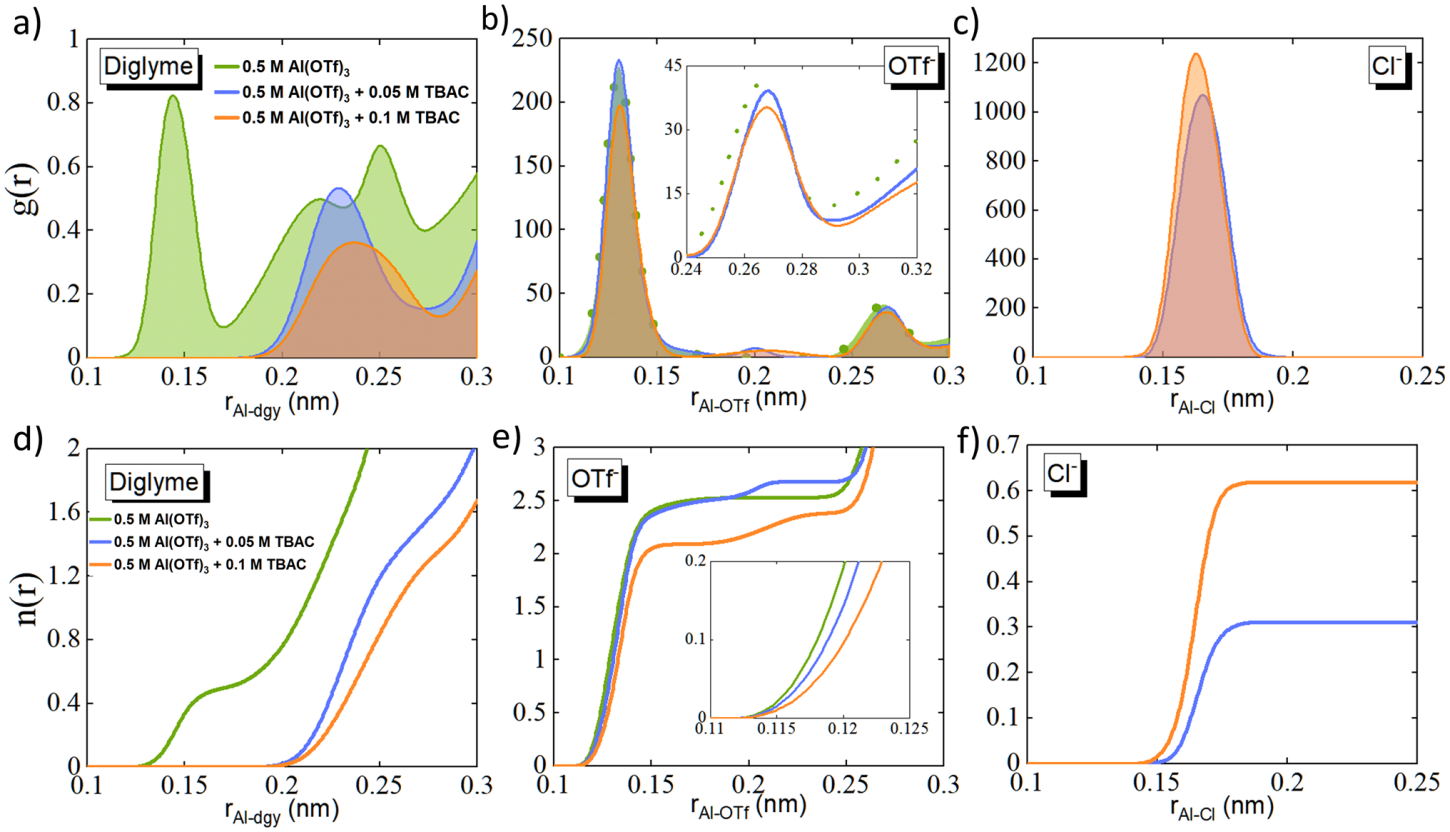
Electrolyte solvation study from molecular dynamics simulations. Radial distribution function (RDF), g(r), of **a** diglyme, **b** OTf^−^ and **c** Cl^−^ as a function of radial distance (r) from Al^3+^ ion for the three electrolyte systems −0.5 M Al(OTf)_3_, 0.5 M Al(OTf)_3_ + 0.05 M TBAC and 0.5 M Al(OTf)_3_ + 0.1 M TBAC. Running coordination number of **d** diglyme, **e** OTf^−^ and **f** Cl^−^ corresponding to (**a**), (**b**) and (**c**), respectively

The evolution of Al-ion solvation sheath as a result of TBAC addition to the electrolyte system with Al(OTf)_3_ salt and diglyme as solvent was studied using MD simulations at an atomistic scale (Fig. S6a, representative simulation box). Three individual systems were modeled with an increasing amount of the additive in the electrolyte i.e., 0.5 M Al(OTf)_3_ + 0 M TBAC, 0.5 M Al(OTf)_3_ + 0.05 M TBAC and 0.5 M Al(OTf)_3_ + 0.1 M TBAC (simulation and computation details are given in the supplementary information). The density distribution profiles of diglyme, OTf^−^, Cl^−^ and TBA^+^ molecules/ions around the Al^3+^ ion were calculated radially using a bin width of 0.05 nm and the results obtained are as shown in Fig. 3a–c and S6b, respectively. The running coordination numbers of all the radial distribution functions (RDFs) described in Fig. 3a–c are presented in Fig. 3d–f. From Fig. 3a, one can observe the changes in the solvation sheaths of Al^3+^ with the addition of TBAC.

**Fig. 4:**
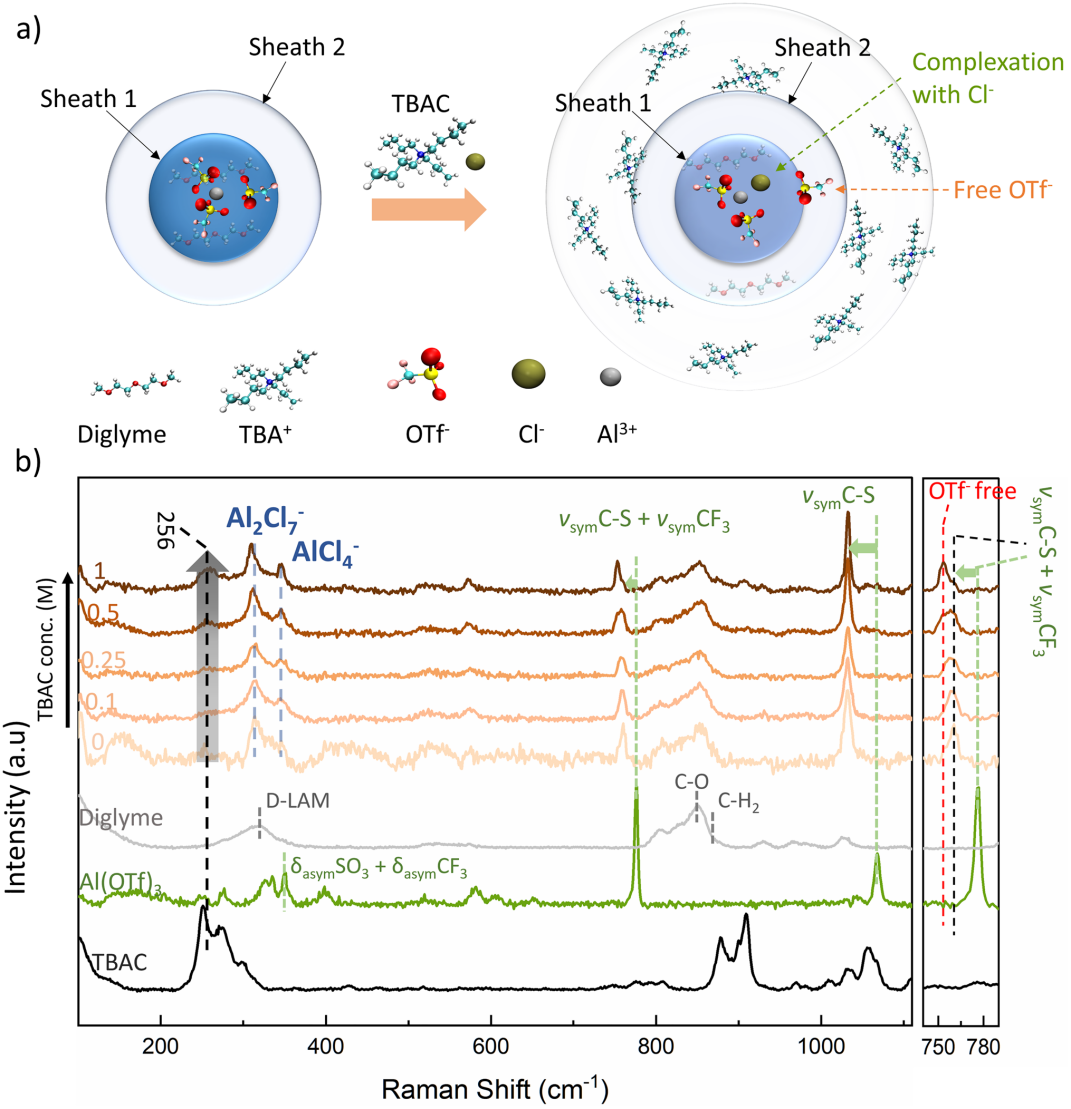
**a** Schematic illustration of the solvation sheath evolution around Al-ion upon TBAC addition into Al(OTf)_3_-diglyme electrolyte (The number of molecules per Al-ion has been rounded off to the closest whole number co-ordination obtained from MD calculations in Figs. 3d–f and S6c). **b** Raman spectra obtained for TBAC, Al(OTf)_3_, diglyme and varying concentrations of TBAC in 0.5 M Al(OTf)_3_ dissolved in diglyme (orange hue)

A considerable decline in the number of diglyme molecules of primary solvation sheath is observed with the addition of 0.05 and 0.1 M TBAC. We can clearly see a shift in the RDF peak of diglyme molecules (Fig. 3a) from 0.12 nm in 0 M TBAC to 0.22 nm in 0.05 M TBAC and 0.23 nm in 0.1 M TBAC, indicating that the diglyme molecules are pushed farther away from the surface of Al^3+^ ions with the increase in TBAC concentration in the electrolyte. The inferences drawn from the diglyme RDF plot are also evident from the coordination number of diglyme molecules as shown in Fig. 3d. Figure 3b depicts the distribution of OTf^−^ ions on Al^3+^ ions. At 0.05 M TBAC concentration, not much decrease in the number of OTf^−^ ions is observed. However, with the addition of 0.1 M TBAC, a considerable decrease in the number of OTf^−^ ions in the primary sheath of Al^3+^ is noticed (clearly seen from Fig. 3e as a decrease in the coordination number of OTf^−^ with Al^3+^). This can be interpreted to be the reduction in the complexation among Al^3+^ and OTf^−^ ions upon TBAC’s addition, thereby availing free OTf^−^ ions in the electrolyte. Figure 3c represents the distribution of Cl^−^ ions around the Al^3+^ ions. Here one can observe an increase in the complexation among Cl^−^ and Al^3+^ ions (at ~ 0.16 nm) with an increase in the TBAC concentration. The same is also evident from the coordination of Cl^−^ ions with Al^3+^ ions (Fig. 3f). Hence, Cl^−^ ions accumulates near the Al^3+^ ions in the primary solvation sheath thereby pushing away the OTf^−^ ions resulting in the reduction in complexation among Al^3+^ ions and OTf^−^ ions. From the RDF profiles of TBA^+^ ions around Al^3+^ ion (Fig. S6b, c), a significant decrease in the distribution of TBA^+^ ions in the vicinity of Al^3+^ ions is seen with the rise in TBAC concentration. This can be because of the increased repulsive nature among the similarly charged ions—Al^3+^ and TBA^+^. Analyzing the MD simulation results, it is clear that the addition of TBAC is causing drastic changes in the immediate vicinity of the Al^3+^ ion (Fig. 4a). Al-ion, which used to be coordinated by OTf^−^ in the immediate vicinity, is now immediately coordinated by Cl^−^ ion as well, essentially indicating the possibility of Al–Cl speciation. There are also fewer OTf^−^ ions in the vicinity of Al^3+^, and some OTf^−^ ions are pushed farther away from the Al^3+^ because of Cl^−^ presence. This also implies the availability of a greater number of free OTf^−^ ions in the electrolyte. At the same time, some solvent molecules are also pushed farther away from Al^3+^ after the addition of TBAC. TBA^+^ ions are mainly present in the outermost solvation sheath.

Further, Raman spectroscopy studies give insight into the complex formation and speciation of the additive modulated electrolyte. The Raman spectrum of the solvent, diglyme, shows a broad band at ~ 316 cm^−1^, corresponding to D-LAM (disordered-longitudinal acoustic mode), which is a combination of C–C–O and C–O–C bending vibrations coupled with torsional C–C motions [46, 47]. This band is sensitive to the nature and the number of conformational states shown by the glyme molecule [46, 47]. Hence, a broad band is expected when glyme is in the pure state, and a narrow band can be expected for a more constricted/complexed diglyme. As expected, upon the addition of Al(OTf)_3_ in diglyme, the D-LAM band narrows, indicating diglyme-OTf complex formation (0 M TBAC in Fig. 4b). D-LAM frequency band further narrows and develops into two distinctive peaks as more TBAC is added to the electrolyte (Fig. 4b), indicating further complexation, this time with TBAC molecule. These two distinctive peaks at 307 and 344 cm^−1^ are well documented in RAB literature and correspond to Al_2_Cl_7_^−^ and AlCl_4_^−^ anions [6, 48], whose possible formation was also indicated in the MD study. Similar observations have also been made in the past for Li salts dissolved in diglyme [46].

The Raman data of Al(OTf)_3_ electrolyte show the characteristic OTf^−^ peaks at 350, 775 and 1067 cm^−1^, corresponding to δ_asym_SO_3_ and δ_asym_ CF_3_, *v*_sym_CF_3_ and *v*_sym_C-S and *v*_sym_ CS signals, respectively [30]. All these peaks show a red shift upon dissolving Al(OTf)_3_ in the diglyme, indicating complete dissolution. As we add TBAC into the electrolyte, the shifted peak at 775 cm^−1^ starts to broaden and further splits into two peaks at 752 and 760 cm^−1^ (zoomed in part of Fig. 4b), which corresponds to the *v*_sym_CF_3_ + *v*_sym_C–S signal from Al(OTf)_3_ and free OTf^−^ ion, respectively [30]. This peak split indicates that the addition of more TBAC is leading to enhanced dissociation of Al(OTf)_3_, which eventually leads to the generation of extra AlCl_4_^−^ (as also evident from the AlCl_4_^−^ peak at 344 cm^−1^ at higher TBAC concentration) and more free OTf^−^ ions. Presence of ample free OTf^−^ ions was also indicated from our MD simulation study and is advantageous as these anions are not bounded and can preferably decompose to form part of the SEI layer. Lastly, a broad peak at 256 cm^−1^ appears as TBAC concentration reaches 1 M. Such peak may correspond to Cl^−^ complexations [30].

### Solid Electrolyte Interphase on Anode

**Fig. 5:**
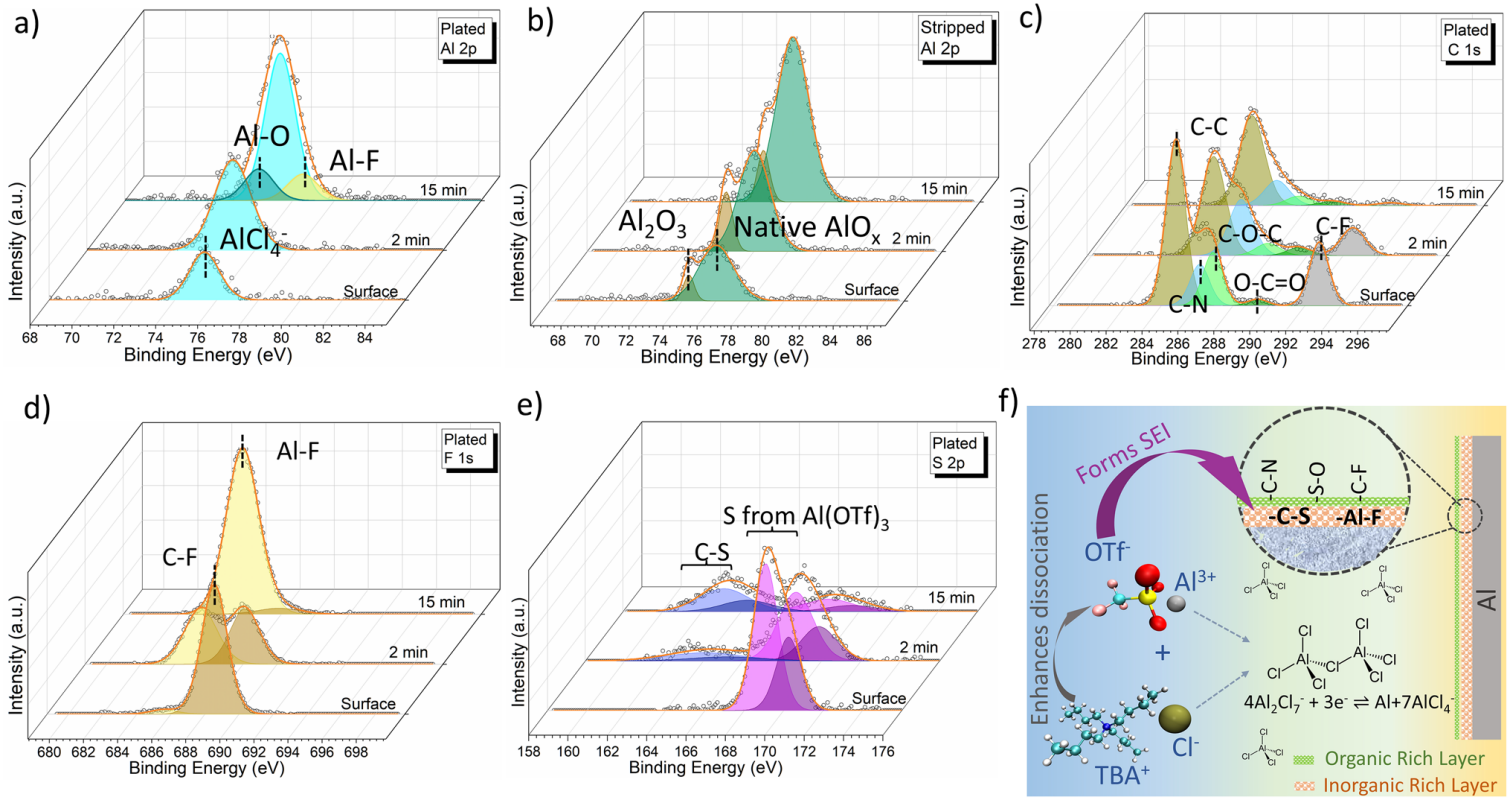
XPS spectra collected from different depths (top surface, after 2 min of etching and after 15 min of etching) of cycled Al-metal in Al symmetric cell: **a** Al 2*p*, **c** C 1*s*, **d** F 1*s* and **e** S 2*p* region of XPS spectra for the plated Al-metal foil. **b** Al 2*p* region of XPS spectra for the stripped Al-metal foil. **f** Schematic illustration of the process steps involved in the modification of the electrolyte speciation upon addition of TBAC in the solvent; the process steps involved in the formation of SEI layer; the composition of the SEI layer

Detailed XPS investigation of the cycled Al metal surfaces and the layers beneath reveals the reaction mechanism at the anode and the formation of an *in-situ* SEI layer during plating/stripping. Figure S7a, b represents the atomic weight percentage of various elements for the plated and stripped Al, respectively, as a function of sputtering depth. It can be seen that the inner layers of the cycled electrodes are not merely composed of Al or its oxides. Rather, it is a composition of species containing elements like F, S and C. The exact nature of these species is further explored based on the energy of the deconvoluted peaks of the spectra. Figures S7(c–f), S8 and S9 represent the layer-wise raw XPS data collected in different elemental regions for the plated or the stripped Al metal samples collected from Al symmetric cells, using Ar^+^ etching to perform depth profiling. Figure 5a–e represents selective data from Figs. S7(c–f), S8 and S9, with deconvoluted peaks for the surfaces after 0, 2 and 15 min of etching.

The Al 2*p* region of the XPS spectrum shows the major presence of AlCl_4_^−^ on the plated sample surface and beneath (Figs. 5a and S7c) [12]. However, for the stripped sample, majorly, oxides of Al are present on the surface and beneath (Figs. 5b and S7e) [49–54]. It can be noted here that we did not see the presence of any metallic Al peak, which typically occurs at 72.7 eV [55, 56]. This can be because of the presence of thick oxide and Cl-rich layer on the top of metallic Al.

Presence and absence of AlCl_4_^−^ on the surface of plated and stripped samples, respectively, is also supported by the presence of metallic Cl and organic Cl (Fig. S7d, f, respectively) on the plated and the stripped sample, respectively [57]. This noticeable difference indicates that it is likely the chlorinated complex of Al that is responsible for charge transfer at the anode. Combining the analysis from the Raman study that Al_2_Cl_7_^−^ and AlCl_4_^−^ are generated when TBAC is added in the electrolyte, and considering the detection of AlCl_4_^−^ on the plated Al surface, we propose the following charge transfer mechanism at the anode:

Anode:

2 $$4{\mathrm{Al}}_{2}{\mathrm{Cl}}_{7}^{-}+3{\mathrm{e}}^{-}\rightleftharpoons \mathrm{Al}+7{\mathrm{AlCl}}_{4}^{-}$$

We speculate that Al_2_Cl_7_^−^ generated upon addition of TBAC into the electrolyte accepts electrons at the anode surface and finally reduces into Al and AlCl_4_^−^ (Eq. 1).

Further investigation of the plated and stripped metal surfaces reveals the composition of the SEI layer. First, we look at the C 1*s* spectrum for the plated sample (Figs. 5c and S8a). The plated surface contains C–C, C–O–C and O–C = O group from the adventitious carbon; C–N group from TBAC; and C–F group from Al(OTf)_3_ [57–59]. Upon etching, the C–N group remains partially intact (also indicated by the presence of *N* (Fig. S8b [60, 61]); however, the C-F group diminishes completely, indicating that C–F is from the electrolyte and does not form the bulk of the SEI. F 1*s* spectrum also supports the presence of C–F only on the surface, indicated by the presence of organic F only on the surface (Figs. 5d and S8c) [62, 63]. Interestingly, the bulk F is metallic, revealing the presence of an Al–F like species [5, 62, 63]. Further, the S 2*p* region (Figs. 5e and S8d) reveals the presence of S from Al(OTf)_3_ on the surface [64], which, however, diminishes upon etching, only to reveal the presence of carbon bonded S (C–S) at a lower energy (different from the energy of SO_3_–C) [60]. The XPS study clearly reveals that Al–F and C–S, which are not originally present in the electrolyte, form part of the bulk SEI. These species would have formed upon electrolyte decomposition of free OTf^−^ anion, which was also detected in the Raman study. The presence of C-N only until a few etched layers indicates that C-N is from the TBAC in the electrolyte and may not form the bulk of the SEI.

Very similar speciation is accounted for when analyzing equivalent XPS data of the stripped sample (Fig. S9). Again, Al–F and C–S are present conspicuously upon etching the top layers, indicating that Al–F and C–S are present in the bulk Al permanently and not merely getting plated/stripped during charge/discharge. In summary, SEI formed on Al-metal can be thought of two different layers—a thin organic-rich layer on the top which is majorly composed of species, C–N, S–O and C–F from the electrolyte and a thick inorganic-rich layer just beneath, which is majorly composed of C-S and Al-F from the decomposition of OTf^−^ (Fig. 5f).

We highlight two important points here explaining why TBAC and Al(OTf)_3_ are a great combination: (1) TBAC is indirectly assisting in the SEI formation. We had seen in the MD and Raman analysis that TBAC augments the dissociation of Al(OTf)_3_ and the generation of free OTf^−^. We speculate that it is these free OTf^−^ that contributes majorly to the SEI formation and (2) replacement of the conventionally used AlCl_3_ with Al(OTf)_3_ not only helps in getting rid of the corrosion problem but also helps in anodic surface protection as Al(OTf)_3_ provides the major SEI bulk components. Notably, using AlCl_3_ as the main salt might not have led to the formation of such SEI layer as it lacks species like F, S and C.

### Full-Cell

**Fig. 6:**
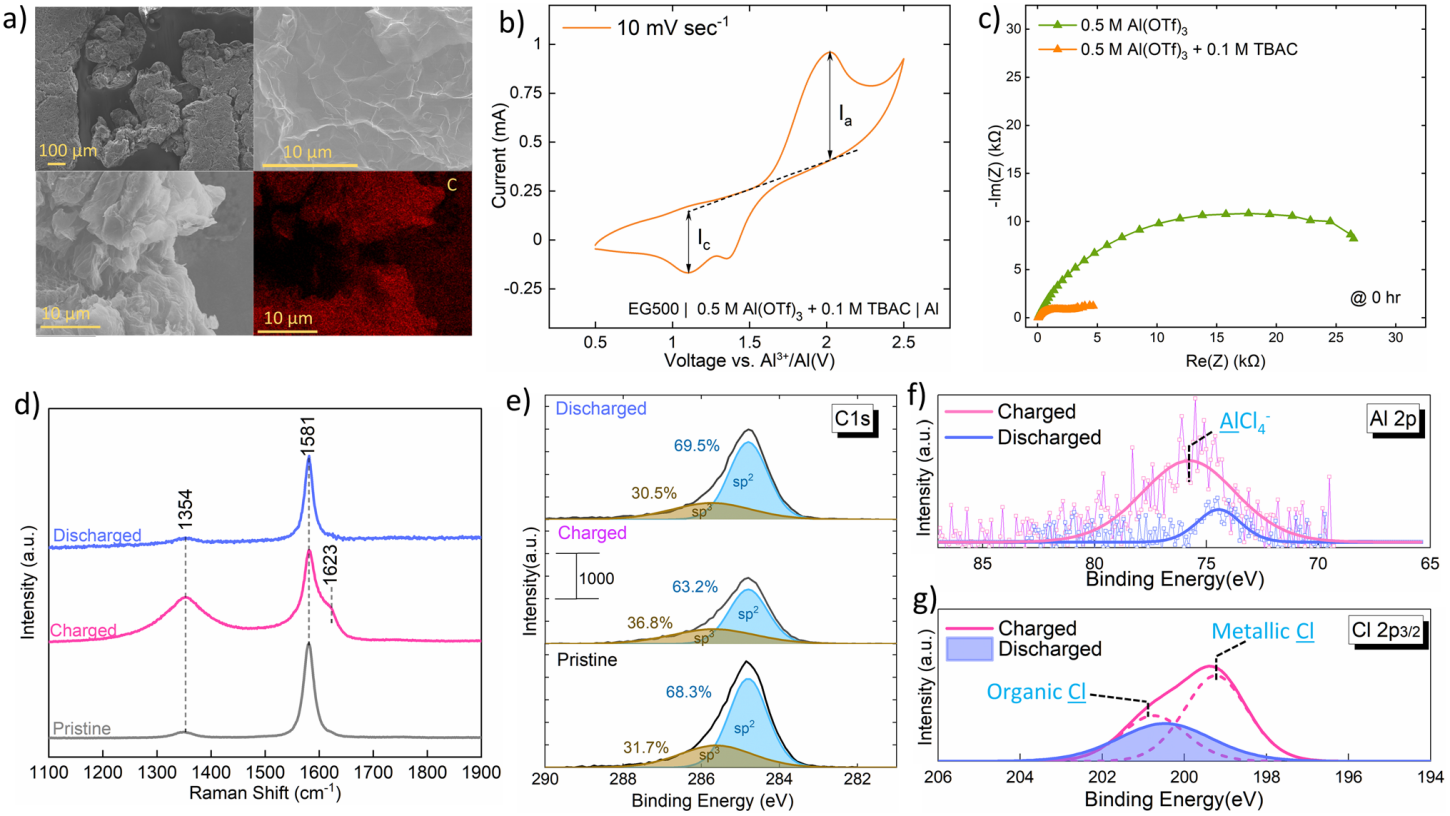
**a** SEM micrograph and EDX map of commercially obtained EG500. **b** Cyclic voltammogram of full-cell—EG500 | 0.5 M Al(OTf)_3_ + 0.1 M TBAC | Al at 10 mV sec.^−1^. **c** Nyquist plots collected for Al symmetric cell, comparing 0.5 M Al(OTf)_3_ and 0.5 M Al(OTf)_3_ + 0.1 M TBAC right after cell fabrication. **d** Ex-situ Raman spectra for pristine, charged and discharged EG500. **e** C 1*s* region of XPS spectra for the pristine, charged and discharged EG500. **f** Al 2*p* and **g** Cl 2*p* region of XPS spectra for the charged and the discharged EG500

The use case of our newly developed electrolyte is shown by fabricating full-cell using commercially obtained expanded graphite-EG500 as the cathode. The obtained expanded graphite is porous and layered (Fig. 6a), which is ideal for improved kinetics and increases the possibility of Al-ion complex insertion.

CV curves (Fig. 6b) corresponding to charge and discharge of EG500 indicate reversible activity in the 0.5 M Al(OTf)_3_ + 0.1 M TBAC electrolyte. Based on our study of electrolyte speciation and reactions at the anode (Eq. 2), it is expected that AlCl_4_^−^ will intercalate in the cathode during the charging and de-intercalate during the discharge process. However, this insertion process is observed to be only partially reversible, as indicated by a dampened cathodic current (*I*_c_/*I*_a_ = 0.58 in Fig. 6b). To probe the role of Cl^−^ in the intercalation/de-intercalation process, we also did a full-cell CV study for different concentrations of TBAC in the electrolyte (Fig. S10a, b). A relatively flat CV profile with no prominent peaks was observed for 0 M TBAC concentration in the 0.5 M Al(OTf)_3_ electrolyte. The absence of redox peaks indicates a negligible contribution of Al(OTf)_3_ to the cathodic capacity. Characteristic redox peaks start to appear as the concentration of TBAC is increased to 0.05 M and further intensifies as the concentration increases to 0.1 M, confirming the active role of Cl^−^ ion in the reaction at the cathode. However, these characteristic CV peaks disappear for higher concentrations of 0.25, 0.5 and 1 M TBAC. We relate the absence of peaks at the high TBAC concentration (≥ 0.25 M) to the high anodic overpotential observed for 0.25, 0.5 and 1 M TBAC (Fig. S1d). We speculate that due to the high anodic overpotential, Al_2_Cl_7_^−^ might not reduce at the anode, hence not producing the AlCl_4_^−^, which would have otherwise replenished the prospect intercalating AlCl_4_^−^ ions in the electrolyte. It can also be noted here that the characteristic redox peaks observed in 0.5 M Al(OTf)_3_ + 0.1 M TBAC electrolyte stabilize only after the first few cycles of CV (Fig. S10c). This observation, which is also highly reproducible when several full-cells were tested, indicates an initial cathode activation. At this stage, we are unable to run highly reversible galvanostatic charge–discharge and attribute it to the inability to control the initial cathode activation step. Nevertheless, CV data provide evidence of reversible intercalation. We believe that further optimization of the cathode structure is required to improve the reversibility [65].

The superiority of the electrolyte is also seen from the EIS study comparing the full-cells with and without TBAC. Clearly, the presence of TBAC aids in keeping the overall cell impedance low during the resting stage (Fig. 6c) and even after the cycling (Fig. S10d).

Further proof of intercalation is obtained using ex-situ Raman analysis (Fig. 6d). Upon full charging of the cell, the originally present graphitic G band (at 1581 cm^−1^) splits into two vibration components (1581 and 1623 cm^−1^). This splitting is likely due to the rearrangement of charges once the intercalation happens [66]. The lower frequency component (*E*_2gi_) at 1581 cm^−1^ corresponds to the vibration of carbon atoms from the un-intercalated graphite, and the 1623 cm^−1^ frequency component ((*E*_2gb_)) corresponds to the vibration of carbon atoms from around the intercalated graphite [67]. Further, upon discharge, the high-frequency peak disappears, revealing reversible de-intercalation of Al complex, at least at a local level. Very similar observations have been made from the in-situ Raman study of graphite in urea + AlCl_3_ and EG500 in triethylamine hydrochloride + AlCl_3_ electrolyte [66, 67]. We also highlight here that no peak shifts were seen upon charging in the ex-situ XRD (Fig. S10e), indicating that the structural changes are local and affecting only the short-range order.

Ex-situ XPS study was performed to decipher the chemical nature of the intercalated species. A 5 min etching was done to get rid of the surface contaminations, including species from the electrolyte. Upon charging the pristine EG500, the weightage of *sp*^3^ components of the C 1*s* peak was observed to increase (Fig. 6e), indicating the oxidation of C upon intercalation. Upon subsequent discharge, the weightage of components shifted back to similar ratios as in the pristine EG500. At the same time, Al 2*p* spectra showed the presence of chlorinated-Al in the charged sample and its absence in the discharged sample (Fig. 6f). The presence of AlCl_4_^−^ is also supported by the detection of metallic Cl component in the charged sample. Such metallic Cl was absent in the discharged sample (Fig. 6g). Al 2*p* and Cl 2*p* spectra indicate the presence of Al–Cl species [12, 68], likely to be AlCl_4_^−^ as reported widely in RAB [15, 69]. We also did an EDX analysis comparing the distribution of Al and Cl on the charged EG500 (Fig. S11a) vs. the discharged EG500 (Fig. S11b). It can be seen that the charged EG500 has a denser distribution of Al and Cl; however, for the discharged EG500, the distribution of Al and Cl is scattered. Using EDX, we also found that Cl to C ratio in the charged sample is 0.011, as compared to 0.0006 in the discharged sample. Similarly, the ratio of Al to C in the charged EG500 was 0.13, as compared to a lower value of 0.004 in the discharged EG500. Raman, XPS and EDX analysis, supported by CV study for varying concentrations of TBAC in the electrolyte, indicates that TBAC is actively involved in contributing toward the activity of EG500. We propose the following cathodic and full-cell reactions. Interestingly, these reactions are the same as what has been widely proposed in previous RAB work that uses AlCl_3_ as the main salt [7, 15, 70–75]. However, our work stands out as, unlike most of these reports, we do not use AlCl_3_ as the main electrolyte salt. We point out that detection of traditional reaction mechanisms also implies compatibility of our novel electrolyte with the previously reported cathode materials.

Cathode:

3 $${\mathrm{AlCl}}_{4}^{-}+{C}_{x}\rightleftharpoons {\mathrm{e}}^{-}+{C}_{x}[{\mathrm{AlCl}}_{4}]$$

Overall:

4 $$4{\mathrm{Al}}_{2}{\mathrm{Cl}}_{7}^{-}+3{C}_{x}\rightleftharpoons 3{C}_{x}\left[{\mathrm{AlCl}}_{4}\right]+\mathrm{Al}+4{\mathrm{AlCl}}_{4}^{-}$$

## Conclusions

In this study, we solve a long-existing problem of using highly corrosive AlCl_3_ salt-based electrolytes in the RAB systems. To address this issue, we report a unique combination of Al(OTf)_3_ salt modulated with TBAC additive, which works in tandem to: (1) generate the required charge carrying species in the electrolyte, (2) activate a rather passivated Al anode surface and (3) protect the Al anode surface during cycling. Using MD simulation, we predict a modified Al-ion solvation sheath as a result of TBAC addition in the Al(OTf)_3_ only electrolyte. Presence of TBAC generates Al_2_Cl_7_^−^, which reduces on the anode surface to AlCl_4_^−^, forming the basic reaction mechanism at the anode. The generation of aluminated chloride species also reduces the charge transfer resistance and surface activation energy at the anode surface, implications of which are seen in a 20-fold reduction in plating/stripping overpotential. We also decipher the formation of fluorine and sulfur rich in-situ SEI layer on the Al-anode surface, which is crucial in protecting the Al-anode from severe oxidation during cycling.

We find our electrolyte above par in terms of anodic overpotential and cycling life when compared with the non-AlCl_3_ electrolytes reported for RAB to date (Table S7). Comparison with AlCl_3_-based electrolytes reveals comparable anodic overpotential and unprecedented plating/stripping cycling life of 1300 cycles for our electrolyte (Table S7). We believe that this study is important in two major ways: (1) electrolyte optimization backed by speciation and surface interphase study will set a roadmap for electrolyte fabrication in similar metal-anode systems like Mg and Zn batteries and (2) this unique combination of Al(OTf)_3_ + TBAC not only enables us to achieve a low anodic overpotential but is also potentially attractive for commercialization because of its high flashpoint, corrosion-free nature and cost-effectiveness.

### Supplementary Information

Below is the link to the electronic supplementary material.

Supplementary file1 (PDF 2344 kb)
