# Supplementary material for: Additive-Driven Interfacial Engineering of Aluminum Metal Anode for Ultralong Cycling Life
Source: Nanomicro Lett. 2022 Dec 29;15:21. doi: 10.1007/s40820-022-01000-6 (PMC9800684; doi:10.1007/s40820-022-01000-6)
Supplement: Supplementary file 1 — Supplementary file1 (PDF 2344 kb) [file 40820_2022_1000_MOESM1_ESM.pdf]

Supporting Information for

Additive-Driven Interfacial Engineering of Aluminum Metal Anode for Ultralong Cycling Life

Sonal Kumar [1], Prasad Rama[2], Gaoliang Yang [1], Wei Ying Lieu [1,3], Deviprasath Chinnadurai [1], Zhi Wei Seh[1,*]

[1]Institute of Materials Research and Engineering, Agency for Science, Technology and Research (A*STAR), 2 Fusionopolis Way, Innovis, 138634, Singapore

[2]Department of Chemistry and Molecular Biology, University of Gothenburg, Gothenburg, 41125, Sweden

[3]Pillar of Engineering Product Development, Singapore University of Technology and Design, 8 Somapah Road, 487372, Singapore

*Corresponding author. E-mail: sehzw@imre.a-star.edu.sg (Zhi Wei Seh)

Supplementary Figures and Tables

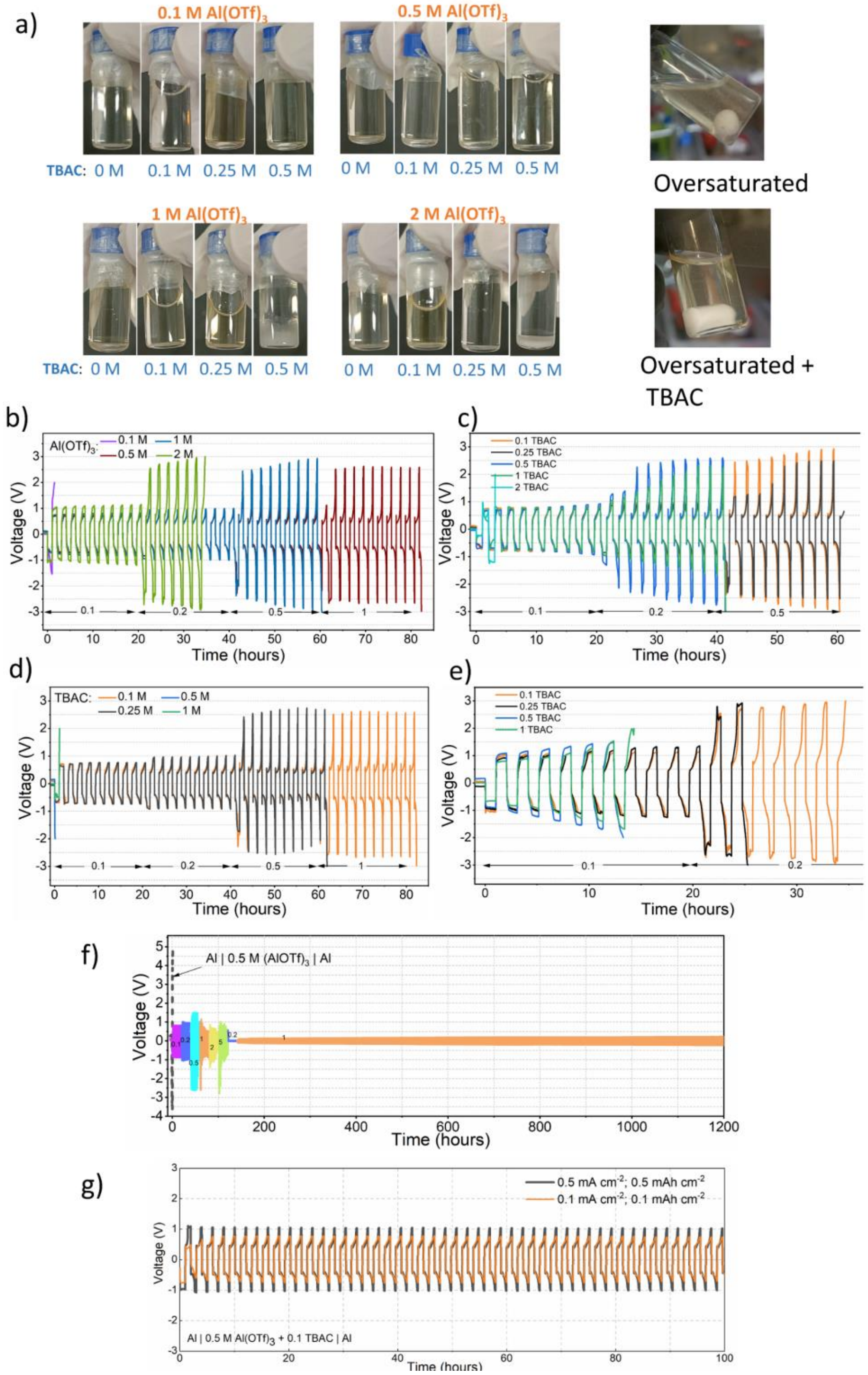

Fig. S1 (**a**) Snapshots of various Al(OTf)$_3$ + TBAC electrolyte configurations used in this study. Fig. on right shows an oversaturated diglyme with Al(OTf)$_3$ and the same solution when TBAC is added into it. Plating/stripping study in symmetric cells at multiple current densities of 0.1, 0.2, 0.5 and 1 mA cm^{-2} for 1 hr each with varying concentrations of: (**b**) Al(OTf)$_3$ + 0.1 M TBAC, (**c**) TBAC + 0.25 M Al(OTf)$_3$, (**d**) TBAC + 0.5 M Al(OTf)$_3$ and (**e**) TBAC + 1 M Al(OTf)$_3$. (**f**) Long term comparative plating/stripping study in 0.5 M Al(OTf)$_3$ vs. 0.5 M Al(OTf)$_3$ + 0.1 M TBAC at multiple current densities, *x*, as indicated in the Fig. (*x* mA cm^{-2}, *x* mAh cm^{-2}) (**g**) Comparative plating/stripping in 0.5 M Al(OTf)$_3$ + 0.1 M TBAC at 0.1 mA cm^{-2}, 0.1 mAh cm^{-2} and 0.5 mA cm^{-2}, 0.5 mAh cm^{-2}

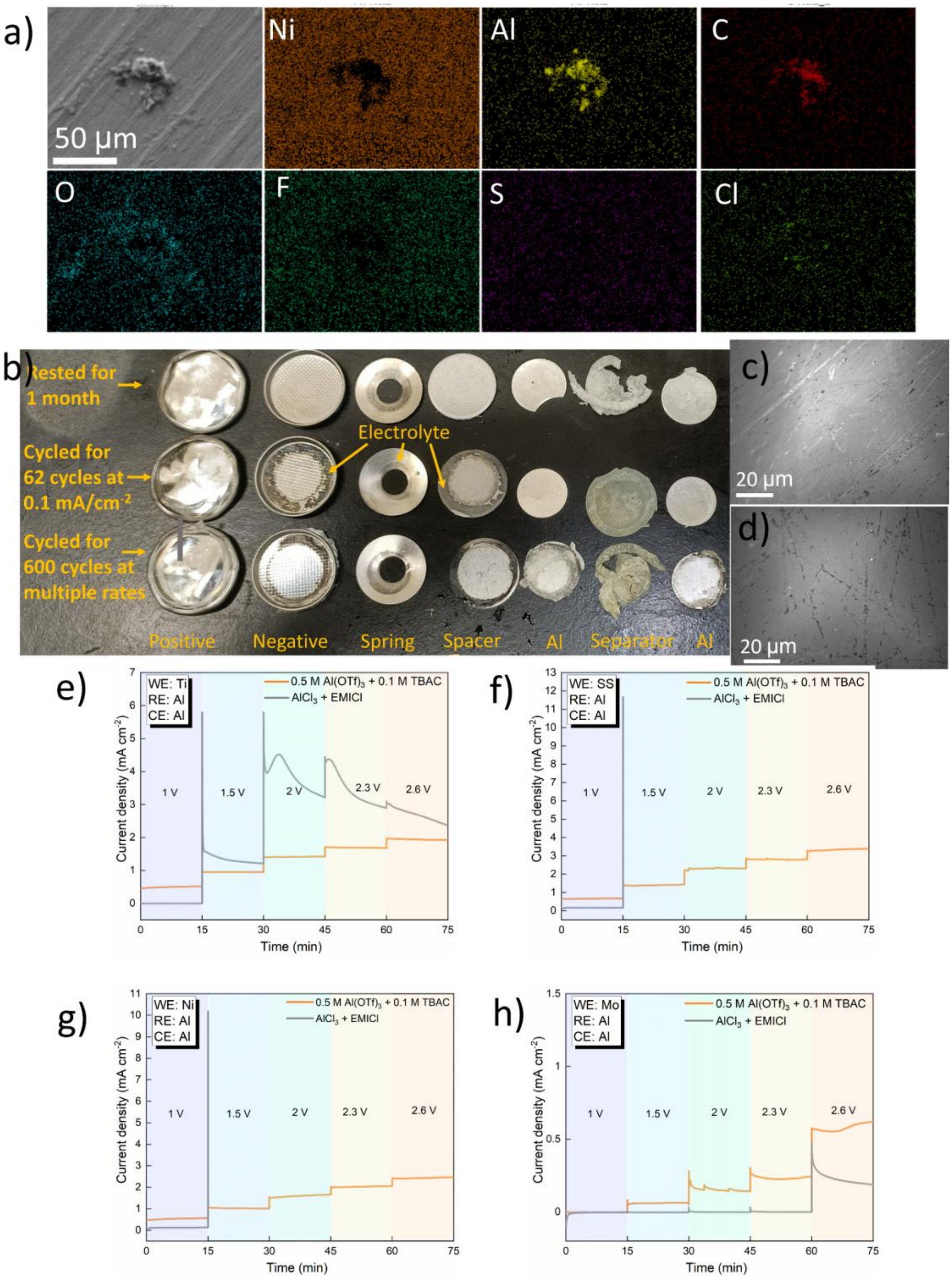

Fig. S2 (**a**) SEM-EDX elemental mappings for Ni, Al, O, F, S, Cl and C on the surface of Al plated Ni foil. The sample is recovered after 5 rounds of plating/stripping (ending with plating) in 0.5 M Al(OTf)$_3$ + 0.1 M TBAC electrolyte at 0.1 mA cm^{-2}, 0.1 mAh cm^{-2}. (**b**) Optical images of cell parts obtained after long periods of resting or cycling to inspect corrosion. SEM micrographs of (**c**) SS spacer polished with 1000 grit sand paper, (**d**) SS spacer polished with 1000 grit sand paper and dipped in ~15 ml of 0.5 M Al(OTf)$_3$ + 0.1 M TBAC electrolyte for ~ 60 hours. The corrosion here is negligible compared to the large corrosion pits typically observed in AlCl$_3$/[BMIM]Cl ionic liquid electrolytes [S1]. Chronoamperometry measurement of 0.5 M Al(OTf)$_3$ + 0.1 M TBAC vs. commercial (Sigma Aldrich – CAS 742872) AlCl$_3$ + EMICl (3:2) electrolyte on (**e**) Ti, (**f**) SS, (**g**) Ni and (**h**) Mo

Table S1 The flash point for various concentrations of TBAC in 0.5 M $Al(OTf)_3$ and its comparison with other commonly used Li-ion battery electrolytes (data adopted from [S2, S3])

**Electrolyte**	**Flash Point (°C)**
This work	
0.5 M $Al(OTf)_3$ + 0 M TBAC in diglyme	59.5
0.5 M $Al(OTf)_3$ + 0.1 M TBAC in diglyme	57.5
0.5 M $Al(OTf)_3$ + 0.25 M TBAC in diglyme	59.5
0.5 M $Al(OTf)_3$ + 0.5 M TBAC in diglyme	56.5
Commonly used Li-ion battery electrolytes	
1 M $LiPF_6$/ EC:EMC (1:1 wt)	31
1 M $LiPF_6$/ EC:DEC (1:1 wt)	38
1 M $LiPF_6$/ PC:DMC(1:1 wt)	26
1 M $LiPF_6$/ EC:DMC(1:1 wt)	25.5
1 M $LiPF_6$/ EC:DMC:EA (1:1:1 wt)	10

Table S2 Electrolyte price comparison for an equivalent weight of 1.264 g

**Chemicals (Purity; CAS)**	**Pricing (in SGD)**	**Total**
1-Ethyl-3-methylimidazolium chloride, EMICl (>99%; 65039-09-0)	555 (25 g)	**Electrolyte 1[S4, S5]**: EMICl:$AlCl_3$ (1:1.3 with total mass of 1.264 g)- **27.24 SGD**
Anhydrous $AlCl_3$ (> 99.99%; 7446-70-0)	525 (25 g)	
Urea (99%; 57-13-6)	40 (25 g)	**Electrolyte 2[S6, S7]**: Urea:$AlCl_3$ (1:1.3 with total mass of 1.264 g)- **20.23 SGD**
Triethylamine hydrochloride (>99%; 554-68-7)	38 (25 g)	**Electrolyte 3[S8]:** Triethylamine hydrochloride: $AlCl_3$ (1:1.5 with total mass of 1.264 g) **-16.61 SGD**
Aluminum trifluoromethanesulfonate, $Al(OTf)_3$ (99.9%; 74974-61-1)	347 (25 g)	**This work**: 0.5 M $Al(OTf)_3$ + 0.1 M TBAC in 1 ml of Diglyme (Total mass-1.264 g)– **5.15 SGD**
Tetrabutylammonium chloride, TBAC (>= 97.0 %; 1112-67-0)	147 (25 g)	
Diethylene glycol dimethyl ether, diglyme (99.5%; 111-96-6)	170 (100 ml)	

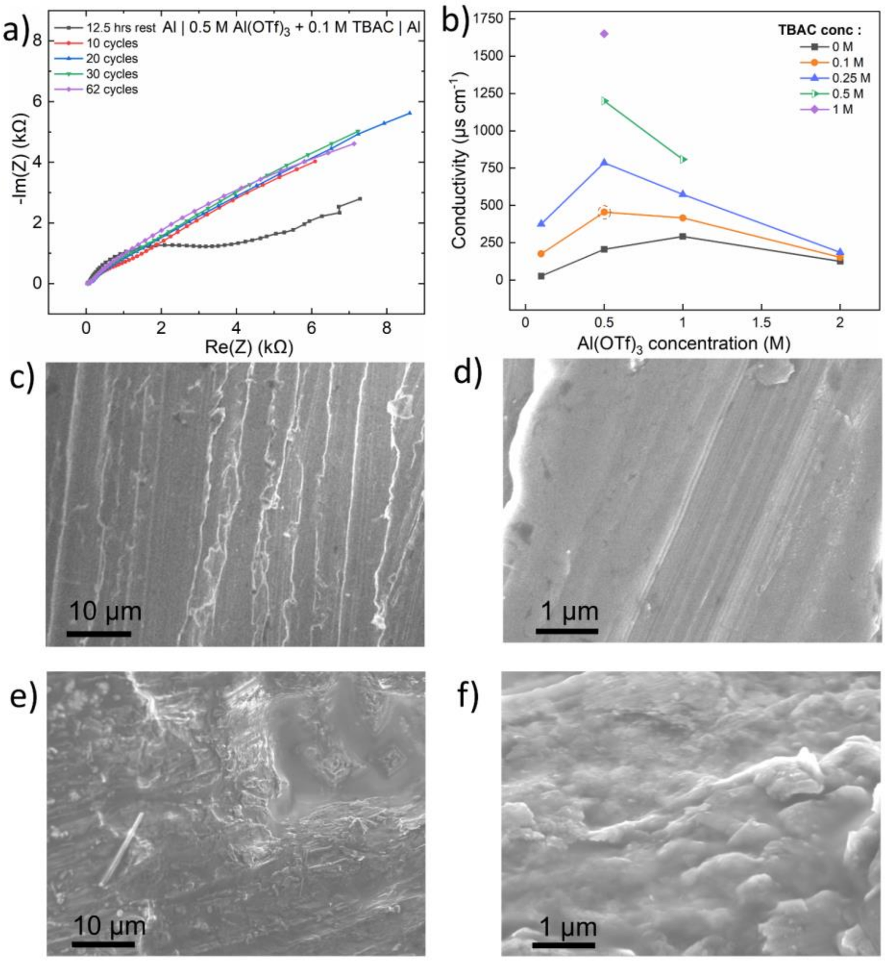

Fig. S3 (**a**) Nyquist plot collected after every 10 cycle of cell fabrication for Al | 0.5 M Al(OTf)$_3$ + 0.1 M TBAC | Al symmetric cells. (**b**) Electrolyte conductivity as a function of Al(OTf)$_3$ concentration with varying amounts of TBAC additive. SEM micrographs of pristine Al at (**c**) 2000 X and (**d**) 20000 X, and plated Al (after 20 cycles; 0.1 mA cm^{-2}, 0.1 mAh cm^{-2}) at (**c**) 2000 X and (**d**) 20000 X

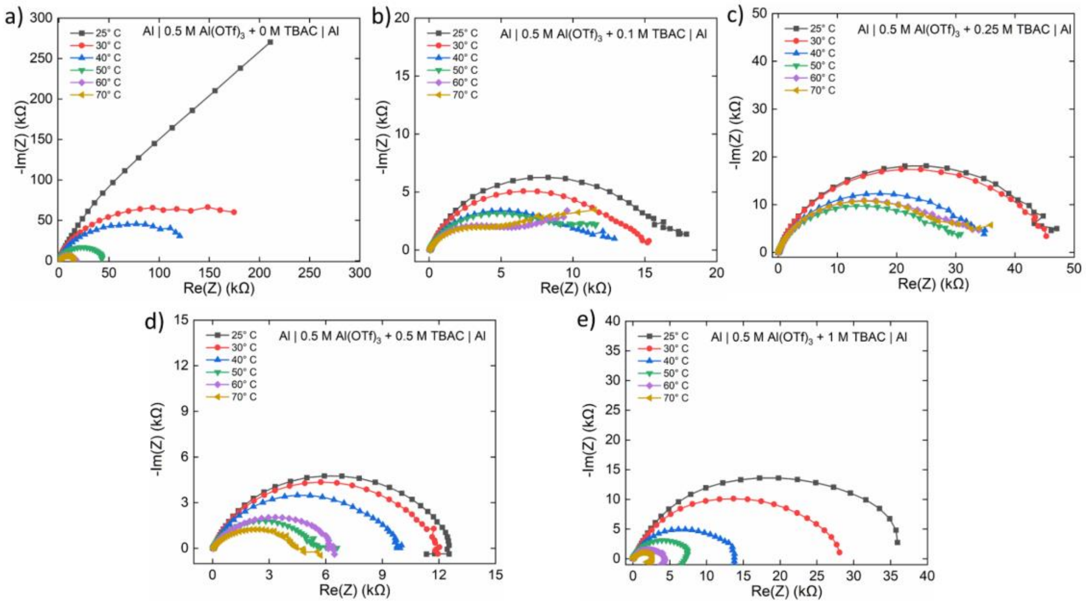

Fig. S4 Nyquist plot collected at various temperatures for varying amounts of TBAC additive in 0.5 M Al(OTf)$_3$ electrolyte: (**a**) 0 M, (**b**) 0.1 M, (**c**) 0.25 M, (**d**) 0.5 M and (**e**) 1 M

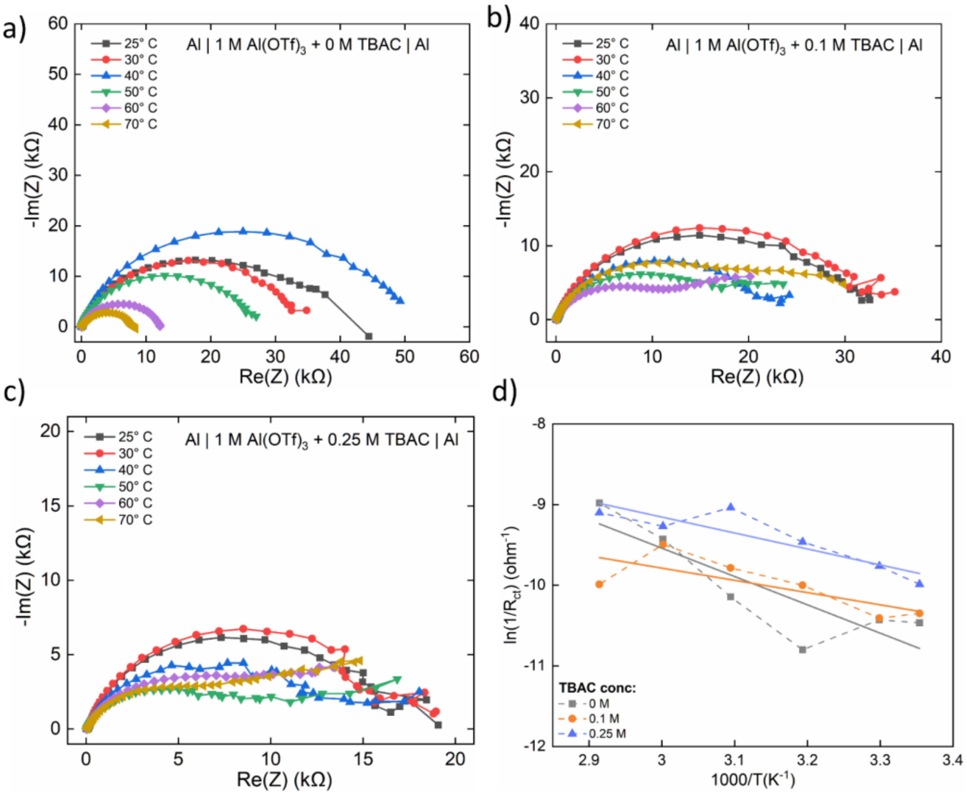

Fig. S5 Nyquist plot collected at various temperatures for varying amounts of TBAC additive in 1 M $Al(OTf)_3$ electrolyte: (**a**) 0 M, (**b**) 0.1 M, (**c**) 0.25 M and (**d**) logarithmic plot of inverse of charge transfer resistance (obtained after fitting respective data in Fig. S5 **a**, **b** and **c**) versus the inverse of the temperature. Each plot, which is also linearly fitted with the solid lines, is for Al | 1 M $Al(OTf)_3$ + *x* M TBAC | Al symmetric cell with varying concentration (*x*) of TBAC

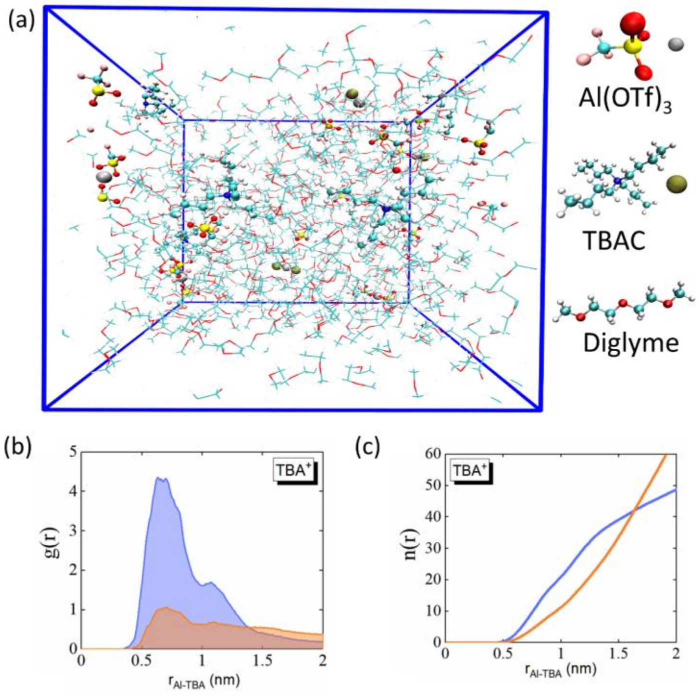

Fig. S6 (**a**) Simulation box with its constituent molecules: diglyme has been shown as stick model in the simulation box. (**b**) Radial distribution and (**c**) calculated co-ordination number of TBA^+ as a function of radial distance from Al^{3+} ion for the two electrolyte systems – 0.5 M $Al(OTf)_3$ + 0.05 M TBAC (purple) and 0.5 M $Al(OTf)_3$ + 0.1 M TBAC (orange)

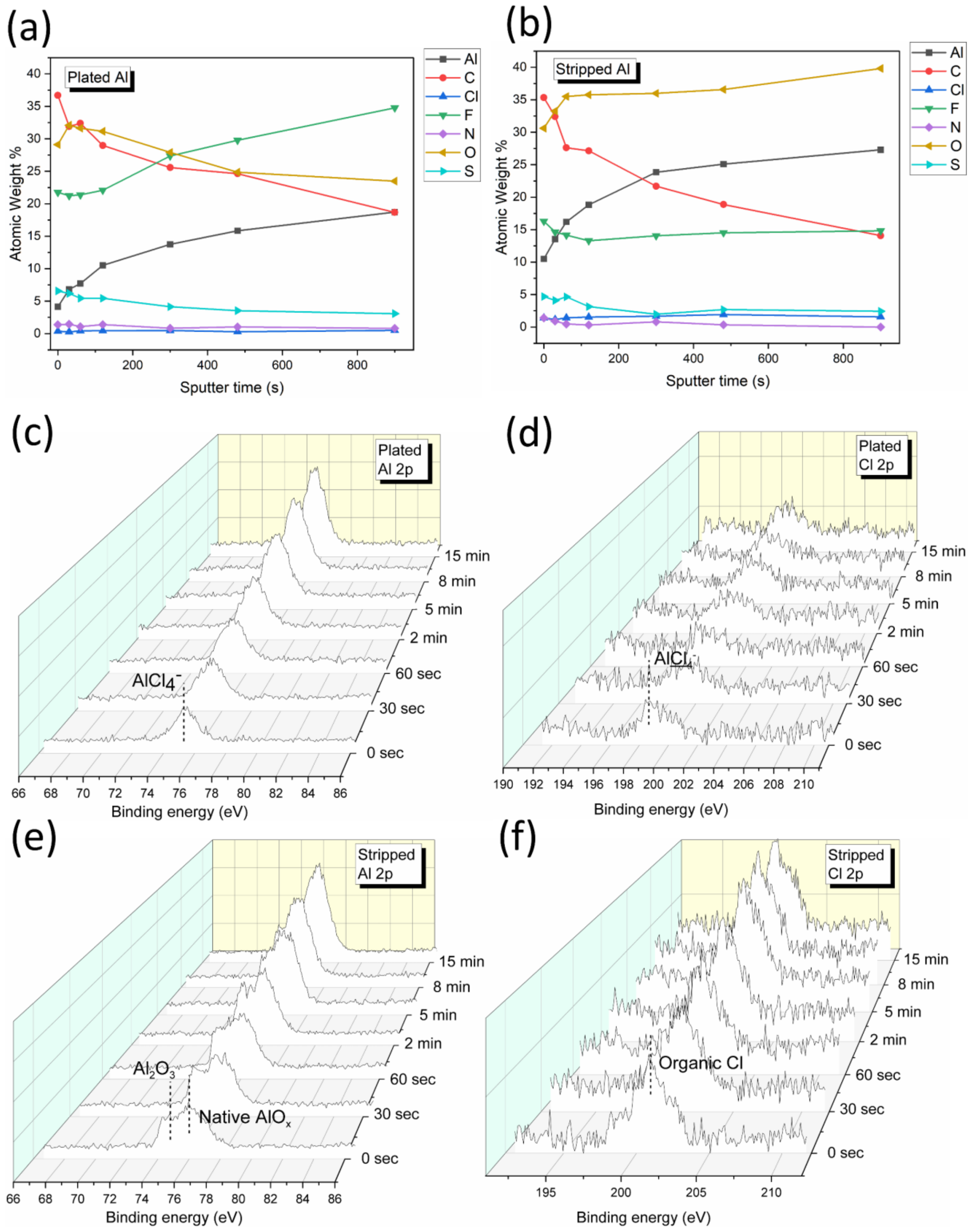

Fig. S7 Atomic weight percentage from the narrow XPS spectrum of the respective elements as a function of sputtering time for the (**a**) plated Al and (**b**) stripped Al retrieved from the cycled symmetric cell. XPS spectra collected from different depths (etching time: 0, 0.5, 1, 2, 5, 8 and 15 min) of cycled Al-metal: (**c**) Al 2p and (**d**) Cl 2p region of XPS spectra for the plated Al-metal foil, and (**e**) Al 2p and (**f**) Cl 2p region of XPS spectra for the stripped Al-metal foil

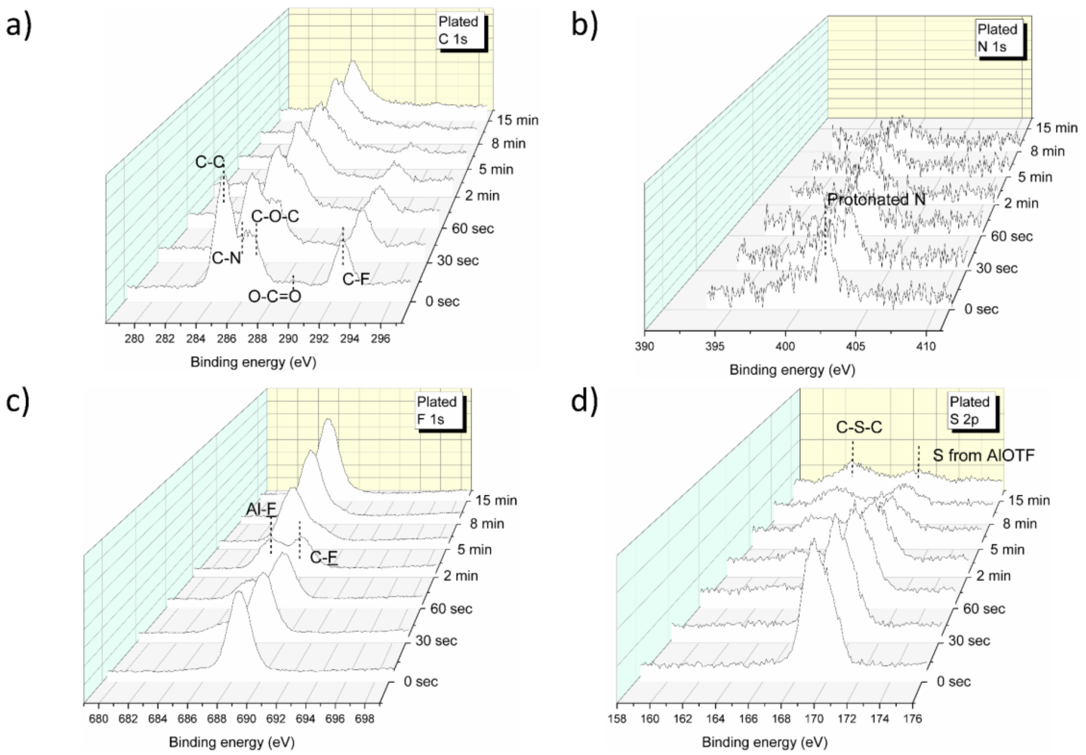

Fig. S8 XPS spectra collected from different depths (etching time: 0, 0.5, 1, 2, 5, 8 and 15 min) of cycled Al-metal: (**a**) C 1s, (**b**) N 1s, (**c**) F 1s and (**d**) S 2p region of XPS spectra for the plated Al-metal foil

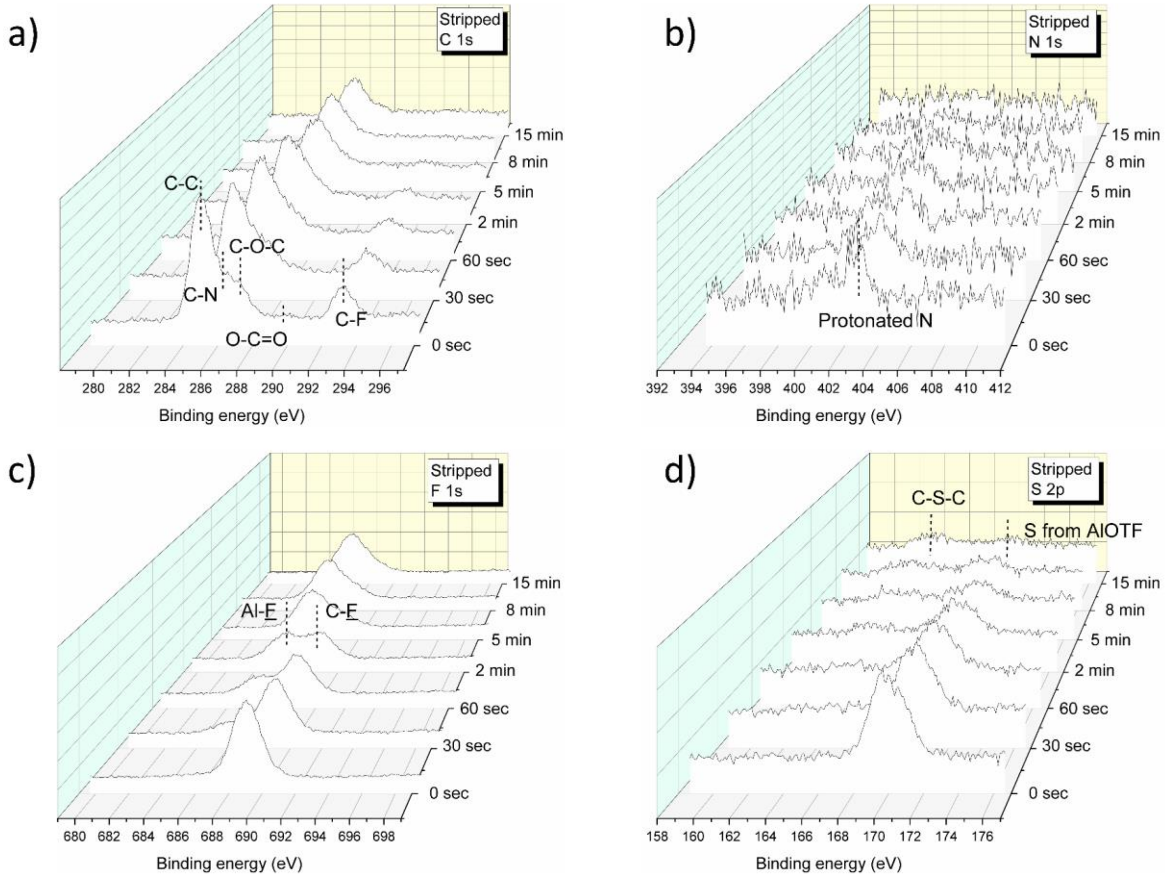

Fig. S9 XPS spectra collected from different depths (etching time: 0, 0.5, 1, 2, 5, 8 and 15 min) of cycled Al-metal: (**a**) C 1s, (**b**) N 1s, (**c**) F 1s and (**d**) S 2p region of XPS spectra for the stripped Al-metal foil

Table S3 Binding energy of species from Al 2p region of XPS spectra discussed in the XPS section

Species	Binding Energy (eV)	Refs.
Metal Al	72.6-72.8	[S9, S10]
Al_2O_3	73.39-74.44	[S11-S14]
$AlCl_4^-$	75.4	[S4]
Native AlO_x	75.29-76.47	[S15, S16]

Table S4 Binding energy of species from O 1s region of XPS spectra discussed in the XPS section

Species	Binding Energy (eV)	Refs.
Al oxide	530.3-531.2	[S17, S18]
Organic species (C=O)	532.30	[S19, S20]
SO_3^-	533.64	[S21]

Table S5 Binding energy of species from C 1s region of XPS spectra discussed in the XPS section

Species	Binding Energy (eV)	Refs.
CF_3	292.8	[S22, S23]
C-N	286.15	[S24]
C-S	285.57	[S25]
O-C=O	289.37	[S26]
C-O-C	286.88	[S26]
C-C	284.8	[S26]

Table S6 Binding energy of various species, F, Cl, S and N as discussed in the XPS section

Species	Binding Energy (eV)	References
C-$\underline{F}$	688.10	[S27, S28]
Al-$\underline{F}$	685.52	[S27, S28]
C-$\underline{Cl}$	200.24	[S26]
$Al\underline{Cl}_4^-$	198.72	[S26]
C-$\underline{S}$	164.30, 163.14	[S29]
$CF_3\underline{S}O_3^-$	169.6, 168.46	[S30]
Protonated $\underline{N}$	402.19	[S31]

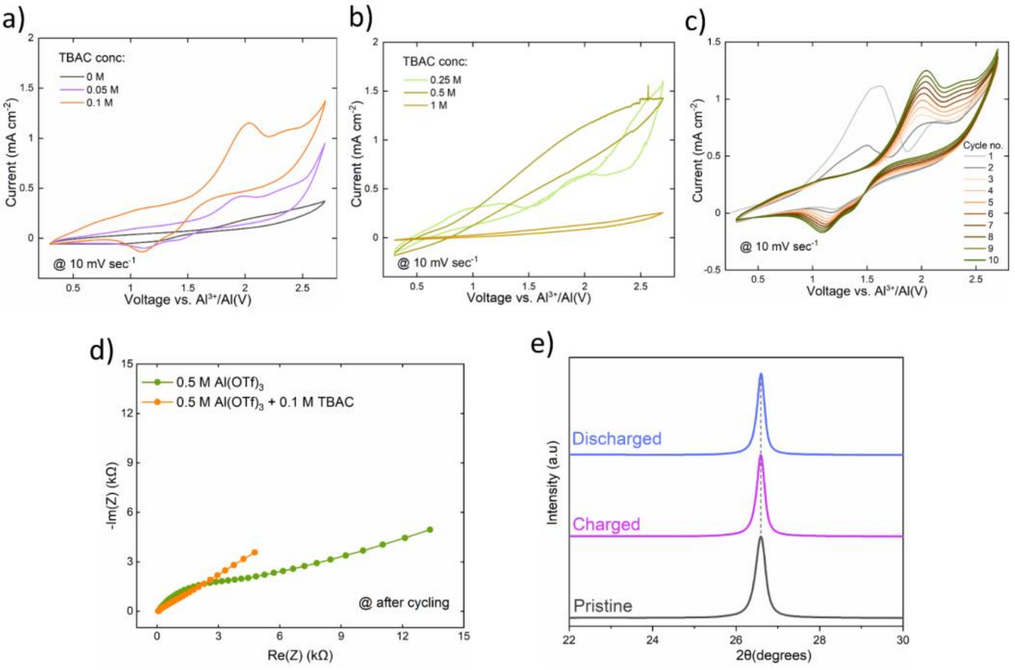

Fig. S10 (**a**) Cyclic voltammogram of full-cell EG500 (8th cycle) at 10 mV sec^{-1} for varying concentrations of TBAC in 0.5 M $Al(OTf)_3$ (**a**) 0 M, 0.05 M, 0.1 M, (**b**) 0.25 M, 0.5 M and 1 M, (**c**) First 10 CV cycles for the full cell – EG500 | 0.5 M $Al(OTf)_3$ + 0.1 M TBAC | Al at 10 mV sec^{-1}, (**d**) Nyquist plots collected for Al symmetric cell, comparing 0.5 M $Al(OTf)_3$ and 0.5 M $Al(OTf)_3$ + 0.1 M TBAC after 5 rounds of cycling. (**b**) X-ray diffraction pattern collected for pristine, charged, and discharged EG 500

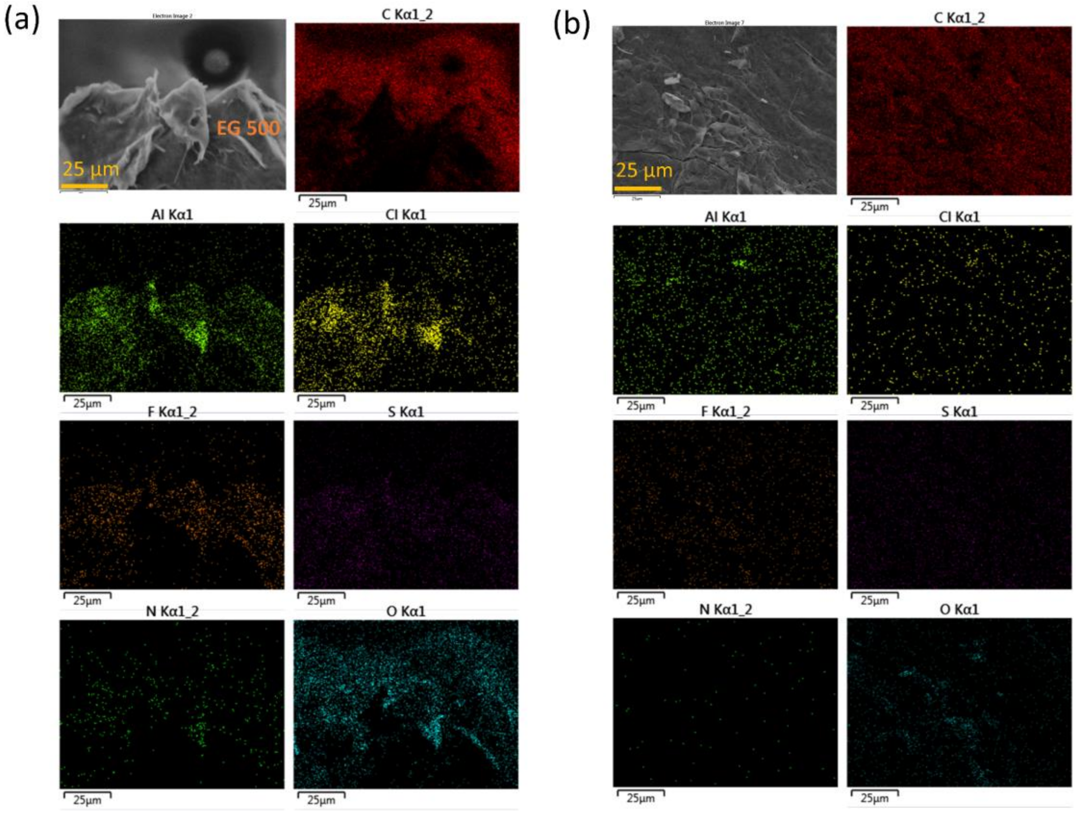

Fig. S11 SEM micrograph and EDX map of (**a**) charged EG500 and (**b**) discharged EG500

Table S7 Table comparing the anodic overpotential and plating/stripping cycling life of our work (in orange) with the non-$AlCl_3$ (in blue) and $AlCl_3$-based (in black) organic electrolytes reported for RAB

No	Electrolyte	Anodic over-potential (stripping)	Cycling rate	Number of cycles (time)	Electro-chemical technique	Refs.
1	0.5 M $Al(OTf)_3$ + 0.1 M TBAC	0.4 V	20 mV s^{-1}	-	CV	This work
2	0.5 M $Al(OTf)_3$ + 0.1 M TBAC	<0.1 V	0.2 mA cm^{-2} , 0.2 mAh cm^{-2}	Tested for 10 cycles (20 h)	Multi-rate GCD	This work
3	0.5 M $Al(OTf)_3$ + 0.1 M TBAC	0.4 V	0.1 mA cm^{-2} , 0.1 mAh cm^{-2}	**1300 cycles (2600 h)**	GCD	This work
4	$[Al(BIm)_6][TFSI]_3$	0.4 V	20 mV s^{-1} (at 80 °C)	-	CV	[S32]
5	0.25 M $Al(PF_6)_3$ with Et_3Al in DMSO	1.2 V	25 mV s^{-1}	-	CV	[S33]
6	0.5 M $Al(TFSI)_3$ in acetonitrile	1 V	10 mV s^{-1}	-	CV	[S34]
7	$Al(OTf)_3$/NMA/urea (molar ratios 0.05/0.76/0.19)	0.6 V	20 mV s^{-1}	-	CV	[S35]
8	NaCl–KCl–$AlCl_3$ (26:13:61, mol/mol)	0.1 V	10 mV s^{-1} (at 180 °C)	-	CV	[S36]
9	NaCl–KCl–$AlCl_3$ (26:13:61, mol/mol)	0.1 V	50 mA cm^{-2} , 1 mAh cm^{-2} (at 180 °C)	50 cycles (2 h)	GCD	[S36]
10	$AlCl_3$/EMIBr =1.3	0.1 V	1 mV s^{-1} (at 80 °C)	-	CV	[S37]
11	$AlCl_3$/[EMIm]Cl =1.3	0.4 V	10 mV s^{-1}	-	CV	[S4]
12	$AlCl_3$/Et_3NHCl = 1.5	0.35 V	1 mV s^{-1}	-	CV	[S38]
13	$AlCl_3$/Et_3NHCl = 1.7 encapsulated in PA	0.3 V	1 mV s^{-1}	-	CV	[S39]
14	$AlCl_3$/Et_3NHCl = 1.5 diluted with organic electrolytes	0.2 V	100 mV s^{-1}	-	CV	[S40]
15	$AlCl_3$/AcAm =1.3	0.07 V	0.2 mA cm^{-2} , 0.2 mAh cm^{-2}	500 cycles (1000 h)	GCD	[S41]
16	PMMA - $AlCl_3$ 0.68 - $EMImAlCl_4$ 80%	0.8 V	50 mV s^{-1}	-	CV	[S42]
17	$AlCl_3$ + EMICl (molar ratio 1.5:1)	0.25 V	20 mV s^{-1}	-	CV	[S35]
18	$AlCl_3$/urea = 1.4	0.28 V	1 mV s^{-1}	-	CV	[S43]
19	$AlCl_3$/urea = 1.3	0.35 V	0.5 mV s^{-1}	-	CV	[S6]
20	$AlCl_3$/[EMIm]Cl =1.3	0.4 V	20 mV s^{-1}	-	CV	[S44]
21	$AlCl_3$/[EMIm]Cl =1.3; with porous Al metal foil	0.25 V	3 mA cm^{-2} , 0.5 mAh cm^{-2}	300 cycles (100 h)	GCD	[S45]

Supplementary References

[S1] H. Wang, S. Gu, Y. Bai, S. Chen, N. Zhu et al., Anion-effects on electrochemical properties of ionic liquid electrolytes for rechargeable aluminum batteries. J. Mater. Chem. A **3**(45), 22677-22686 (2015). https://doi.org/10.1039/C5TA06187C

[S2] S. Hess, M. Wohlfahrt-Mehrens, M. Wachtler, Flammability of Li-ion battery electrolytes: flash point and self-extinguishing time measurements. J. Electrochem. Soc.

162, A3084 (2015). https://doi.org/10.1149/2.0121502jes

[S3] G.G. Eshetu, S. Grugeon, S. Laruelle, S. Boyanov, A. Lecocq et al., In-depth safety-focused analysis of solvents used in electrolytes for large scale lithium ion batteries. Phys. Chem. Chem. Phys. **15**, 9145 (2013). https://doi.org/10.1039/C3CP51315G

[S4] MC. Lin, M. Gong, B. Lu, Y. Wu, D.Y. Wang et al., An ultrafast rechargeable aluminium-ion battery. Nature **520**, 324 (2015). https://doi.org/10.1038/nature14340

[S5] S. Wang, Z. Yu, J. Tu, J. Wang, D. Tian et al., A novel aluminum-ion battery: Al/$AlCl_3$-[EMIm]Cl/Ni_3S_2@graphene. Adv. Energy Mater. **6**(13), 1600137 (2016). https://doi.org/10.1002/aenm.201600137

[S6] M. Angell, C.J. Pan, Y. Rong, C. Yuan, M.C. Lin et al., High coulombic efficiency aluminum-ion battery using an $AlCl_3$-urea ionic liquid analog electrolyte. PNAS **114**, 834 (2017). https://doi.org/10.1073/pnas.1619795114

[S7] M. Elsharkawi, A.M.K. Esawi, Development of an $AlCl_3$-urea ionic liquid for the electroless deposition of aluminum on carbon nanotubes. ACS Omega **5**(11), 5756-5761 (2020). https://doi.org/10.1021/acsomega.9b03805

[S8] X. Dong, H. Xu, H. Chen, L. Wang, J. Wang et al., Commercial expanded graphite as high-performance cathode for low-cost aluminum-ion battery. Carbon **148**, 134-140 (2019). https://doi.org/10.1016/j.carbon.2019.03.080

[S9] R. Hauert, J. Patscheider, M. Tobler, R. Zehringer, XPS investigation of the a-C : H/Al interface. Surf. Sci. **292**, 121 (1993). https://doi.org/10.1016/0039-6028(93)90395-Z

[S10] K. Hirakawa, Y. Hashimoto, T. Ikoma, Orientation independence of heterojunction-band offsets at GaAs-AlAs heterointerfaces characterized by x-ray photoemission spectroscopy. Appl. Phys. Lett. **57**(24), 2555 (1990). https://doi.org/10.1063/1.103815

[S11] Y.C. Kim, H.H. Park, J.S. Chun, W.J. Lee, Compositional and structural analysis of aluminum oxide films prepared by plasma-enhanced chemical vapor deposition. Thin Solid Films **237**, 57-65 (1994). https://doi.org/10.1016/0040-6090(94)90238-0

[S12] Y. Okamoto, K. Nagata, T. Adachi, T. Imanaka, K. Inamura et al., Preparation and characterization of highly dispersed cobalt oxide and sulfide catalysts supported on silica. J. Phys. Chem. **95**(1), 310-319 (1991). https://doi.org/10.1021/j100154a057

[S13] G. Mattogno, G. Righini, G. Montesperelli, E. Traversa, XPS analysis of the interface of ceramic thin films for humidity sensors. Appl. Surf. Sci. **70-71**, 363-366 (1993). https://doi.org/10.1016/0169-4332(93)90459-O

[S14] G. Mattogno, G. Righini, G. Montesperelli, E. Traversa, X-ray photoelectron spectroscopy investigation of $MgAl_2O_4$ thin films for humidity sensors. J. Mater. Res. **9**, 1426-1433 (1994). https://doi.org/10.1557/JMR.1994.1426

[S15] S.L. Chang, J.W. Anderegg, P.A. Thiel, Surface oxidation of an Al-Pd-Mn quasicrystal, characterized by x-ray photoelectron spectroscopy. J. Non-Cryst. Solids **195**, 95 (1996). https://doi.org/10.1016/0022-3093(95)00537-4

[S16] T.J. Carney, P. Tsakiropoulos, J.F. Watts, J.E. Castle, Oxidation and surface segregation in rapidly solidified aluminum alloy powders. Int. J. Rapid Solidification **5**, 189-217 (1990).

[S17] T. L. Barr, The nature of the relative bonding chemistry in zeolites: an XPS study. Zeolites **10**(8), 760-765 (1990). https://doi.org/10.1016/0144-2449(90)90058-Y

[S18] Y. Okamoto, T. Imanaka, S. Teranishi, Surface structure of CoO-$MoO_3Al_2O_3$ catalysts

studied by X-ray photoelectron spectroscopy. J. Catal. **65**, 448 (1980). https://doi.org/10.1016/0021-9517(80)90322-X

[S19] K. Endo, C. Inoue, N. Kobayashi, M. Aida, Spectra analysis of the XPS core and valence energy levels of polymers by an ab initio Mo method using simple model molecules. J. Phys. Chem. Solids **55**, 471 (1994). https://doi.org/10.1016/0022-3697(94)90151-1

[S20] G. Beamson, D. Briggs, High resolution XPS of organic polymers: the scienta ESCA300 database. J. Chem. Educ. **70**(1), A25 (1993). https://doi.org/10.1021/ed070pA25.5

[S21] W. Yao, Z. Zhang, J. Gao, J. Li, J. Xu et al., Vinyl ethylene sulfite as a new additive in propylene carbonate-based electrolyte for lithium ion batteries. Energy Environ. Sci. **2**(10), 1102-1108 (2009). https://doi.org/10.1039/B905162G

[S22] J.C. Lascovich, S. Scaglione, Comparison among XAES, PELS and XPS techniques for evaluation of sp^2 percentage in a-C:H. Appl. Surf. Sci. **78**(1), 17-23 (1994). https://doi.org/10.1016/0169-4332(94)90026-4

[S23] H. Hantsche, High resolution XPS of organic polymers, the scienta ESCA300 database. Adv. Mater. **5**(10), 778 (1993). https://doi.org/10.1002/adma.19930051035

[S24] H. Nie, M. Li, Q. Li, S. Liang, Y. Tan et al., Carbon dots with continuously tunable full-color emission and their application in ratiometric pH sensing. Chem. Mater. **26**(10), 3104 (2014). https://doi.org/10.1021/cm5003669

[S25] J. Peeling, F.E. Hruska, D.M. Mckinnon, M.S. Chauhan, N.S. Mclntyre, ESCA studies of the uracil base. The effect of methylation, thionation, and ionization on charge distribution. Can. J. Chem. **56**(18), 2405-2411 (1978). https://doi.org/10.1139/v78-393

[S26] J.R. Araujo, B.S. Archanjo, K.R. Souza, W. Kwapinski, N.P.S. Falcão et al., Selective extraction of humic acids from an anthropogenic Amazonian dark earth and from a chemically oxidized charcoal. Biol. Fertil. Soils **50**, 1223-1232 (2014). https://doi.org/10.1007/s00374-014-0940-9

[S27] M. Davies, High resolution XPS of organic polymers: the scienta ESCA300 database: G. Beamson and D. Briggs John Wiley, Chichester, UK 1992. Biomaterials **15**, 318 (1994). https://doi.org/10.1016/0142-9612(94)90060-4

[S28] E. Kemnitz, A. Kohne, I. Grohmann, A. Lippitz, W.E.S. Unger, X-ray photoelectron and X-ray excited auger electron spectroscopic analysis of surface modifications of chromia during heterogeneous catalyzed chlorine/fluorine exchange. J. Catal. **159**, 270-279 (1996). https://doi.org/10.1006/jcat.1996.0088

[S29] T. Yoshida, K. Yamasaki, S. Sawada, An X-ray photoelectron spectroscopic study of 2-mercaptobenzothiazole metal complexes. Bull. Chem. Soc. Jpn. **52**, 2908 (1979). https://doi.org/10.1246/bcsj.52.2908

[S30] A.K. Friedman, W. Shi, Y. Losovyj, A.R. Siedle, L.A. Baker, Mapping microscale chemical heterogeneity in Nafion membranes with X-ray photoelectron spectroscopy. J. Electrochem. Soc. **165**, H733 (2018). https://doi.org/10.1149/2.0771811jes

[S31] J. Escard, G. Mavel, J.E. Guerchais, R. Kergoat, X-ray photoelectron spectroscopy study of some metal(II) halide and pseudohalide complexes. Inorg. Chem. **13**(3), 695-701 (1974). https://doi.org/10.1021/ic50133a036

[S32] T. Mandai, P. Johansson, Haloaluminate-free cationic aluminum complexes: structural characterization and physicochemical properties. J. Phys. Chem. C **120**(38), 21285-

21292 (2016). https://doi.org/10.1021/acs.jpcc.6b07235

[S33] X. Wen, J. Zhang, H. Luo, J. Shi, C. Tsay et al., Synthesis and electrochemical properties of aluminum hexafluorophosphate. J. Phys. Chem. **12**(25), 5903-5908 (2021). https://doi.org/10.1021/acs.jpclett.1c01236

[S34] M. Chiku, S. Matsumura, H. Takeda, E. Higuchi, H. Inoue, Aluminum bis(trifluoromethanesulfonyl)imide as a chloride-free electrolyte for rechargeable aluminum batteries. J. Electrochem. Soc. **164**, A1841 (2017). https://doi.org/10.1149/2.0701709jes

[S35] T. Mandai, P. Johansson, Al conductive haloaluminate-free non-aqueous room-temperature electrolytes. J. Mater. Chem. A **3**(23), 12230-12239 (2015). https://doi.org/10.1039/C5TA01760B

[S36] Q. Pang, J. Meng, S. Gupta, X. Hong, C.Y. Kwok et al., Fast-charging aluminium–chalcogen batteries resistant to dendritic shorting. Nature **608**, 704 (2022). https://doi.org/10.1038/s41586-022-04983-9

[S37] H. Yang, L. Yin, J. Liang, Z. Sun, Y. Wang et al., An aluminum–sulfur battery with a fast kinetic response. Angew. Chem. Int. Ed. **57**(7), 1898-1902 (2018). https://doi.org/10.1002/anie.201711328

[S38] H. Xu, T. Bai, H. Chen, F. Guo, J. Xi et al., Low-cost $AlCl_3/Et_3NHCL$ electrolyte for high-performance aluminum-ion battery. Energy Storage Mater. **17**, 38-45 (2019). https://doi.org/10.1016/j.ensm.2018.08.003

[S39] Z. Liu, X. Wang, Z. Liu, S. Zhang, Z. Lv et al., Low-cost gel polymer electrolyte for high-performance aluminum-ion batteries. ACS Appl. Mater. Interfaces **13**(24), 28164-28170 (2021). https://doi.org/10.1021/acsami.1c05476

[S40] S. Xia, X.M. Zhang, K. Huang, Y.L. Chen, Y.T. Wu, Ionic liquid electrolytes for aluminium secondary battery: influence of organic solvents. J. Electroanal. Chem. **757**, 167-175 (2015). https://doi.org/10.1016/j.jelechem.2015.09.022

[S41] L. Zhang, Q. Ma, G. Wang, Z. Liu, L. Zhang, A low cost electrolyte of $AlCl_3$/AcAm ionic liquid analogs for high-performance aluminum ion batteries. J. Electroanal. Chem. **888**, 115176 (2021). https://doi.org/10.1016/j.jelechem.2021.115176

[S42] J.D. Ortiz-Gonzalez, C.I. Sánchez-Sáenz, Gel polymer electrolyte for aluminum-ion batteries. ECS Transact. **100**, 73 (2021). https://doi.org/10.1149/10001.0073ecst

[S43] M. Angell, G. Zhu, M.C. Lin, Y. Rong, H. Dai, Ionic liquid analogs of $AlCl_3$ with urea derivatives as electrolytes for aluminum batteries. Adv. Funct. Mater. **30**(4), 1901928 (2020). https://doi.org/10.1002/adfm.201901928

[S44] H. Go, M.R. Raj, Y. Tak, G. Lee, Electrochemically surface-modified aluminum electrode enabling high performance and ultra-long cycling life Al-ion batteries. Electroanalysis **34**(8), 1308-1317 (2022). https://doi.org/10.1002/elan.202100669

[S45] Y. Long, H. Li, M. Ye, Z. Chen, Z. Wang et al., Suppressing al dendrite growth towards a long-life Al-metal battery. Energy Storage Mater. **34**, 194-202 (2021). https://doi.org/10.1016/j.ensm.2020.09.013
